# Gut microbiota-associated taurine metabolism dysregulation in a mouse model of Parkinson’s disease

**DOI:** 10.1128/msphere.00431-23

**Published:** 2023-10-11

**Authors:** Can Cui, Huan Song, Yingying Han, Hongxiang Yu, Hongxia Li, Yumei Yang, Bei Zhang

**Affiliations:** 1 Department of Neurology, Shanghai East Hospital, School of Medicine, Tongji University, Shanghai, China; 2 Department of Digestive Diseases, Huashan Hospital, Fudan University, Shanghai, China; University of Michigan, Ann Arbor, Michigan, USA

**Keywords:** Parkinson's disease, microbiota, metabolism, taurine, *Lactobacillus*

## Abstract

Parkinson’s disease (PD) is recognized as a multisystem disease concerning gastrointestinal (GI) dysfunction and microbiota dysbiosis. However, the mechanism by which microbial dysbiosis contributes to pathogenesis of PD and the relationship between gut microbes and metabolites remain to be deeply elucidated. This study aims to explore the altered microflora and serum metabolites in PD mice and their role in PD etiology. Herein, 1-methyl-4-phenyl-1,2,3,6-tetrahydropyridine (MPTP, 30 mg/kg) was injected intraperitoneally to establish the PD mice model. 16S rRNA sequencing and ultrahigh-performance liquid chromatography coupled with Q Exactive HF-X mass spectrometry were used to depict the profile of gut bacteria and metabolites. Phylogenetic investigation of communities by reconstruction of unobserved states 2 analysis was performed to elucidate the functional link between the microbe-metabolite axis and PD. We confirmed the MPTP-induced dopaminergic (DA) neuron loss accompanied by the GI dysfunction. Higher abundances of *Aerococcus*, *Staphylococcus*, and *Ruminococcaceae*, and lower abundances of *Lactobacillus*, *Lachnospiraceae*, and *Adlercreutzia* were identified in PD mice. Meanwhile, the differential metabolites concerning “taurine and hypotaurine metabolism” were markedly downregulated in PD mice. Furthermore, *Lactobacillus*, *Adlercreutzia*, and taurine-related metabolites showed the most significant correlation with pathological and GI performance of PD mice. The abundances of microbial transporter and enzymes participating in the degeneration of taurine were disturbed in PD mice. More importantly, taurine supplement is protected from MPTP-induced motor deficits and DA neuron loss. The detection of disturbed taurine metabolism in PD mice suggests a mechanism whereby dysregulation of the microbiota-metabolism axis contributes to the etiology of MPTP-treated mice.

**IMPORTANCE:**

PD is recognized as a multisystem disease concerning GI dysfunction and microbiota dysbiosis but still lacks ideal therapies. Recently, aberrant microbiota-derived metabolites are emerging as important participants in PD etiology. However, the alterations of gut microbiota community and serum untargeted metabolite profile have not been fully investigated in a PD mice model. Here, we discover sharply reduced levels of *Lactobacillus* and taurine in MPTP-treated mice. Moreover, *Lactobacillus*, *Adlercreutzia*, and taurine-related metabolites showed the most significant correlation with pathological and GI performance of PD mice. The abundances of microbial transporter and enzymes participating in the degeneration of taurine were disturbed in PD mice. Most importantly, taurine supplement ameliorates MPTP-induced motor deficits, DA neuron loss, and microglial activation. Our data highlight the impaired taurine-based microbiome-metabolism axis during the progression of PD and reveal a novel and previously unrecognized role of genera in modulating taurine metabolism.

## INTRODUCTION

Parkinson’s disease (PD) is a common neurodegenerative disease characterized by dopaminergic (DA) neuron loss and α-synuclein (αSyn) aggregation in the substantial nigra-striatum system (1). At present, the therapeutic strategy for PD is the classical method that can only ameliorate the motor symptoms and is ineffective in slowing disease progression (2). The etiology of PD remains obscure, but growing evidence indicates that PD is marked as a “body-first” disease that begins in the gut (1, 3). Most PD patients suffer from non-motor deficiency including comorbid gastrointestinal (GI) manifestations such as constipation, sialorrhea, and dysphagia, which can occurred decades before the onset of motor symptoms (2). Accordingly, the enteric nervous system and parasympathetic nerves are among the structures earliest affected by αSyn pathology. More importantly, recent works show that the occurrence of GI symptoms heavily influences the quality of life of PD patients (4). Despite these advances, how the GI tract participates in PD pathogenesis is still not fully understood.

A growing body of research has revealed that intestinal microbiota are not just passive residents, but they also play an important role in modulating vital host physiological systems and act as a bi-directional communicator between gut and brain (1, 5). The intestinal microbiota encompasses trillions of organisms that reside in the lower GI tract and dominates the function of GI (5–8). Aberrant gut microbiota composition and function, termed dysbiosis, are suggested to be a consistent feature of neurodegenerative diseases including PD (9). Both clinical and preclinical studies demonstrated that fecal and mucosa-derived gut microbiota are different between PD and healthy controls (10, 11). Moreover, depletion of the microbiome has been shown to affect progression of PD in a mice model. Antibiotic-treated mice attenuated DA neuron loss and motor deficits and downregulated expression of pro-inflammatory markers including interleukin (IL)-1β and tumor necrosis factor alpha (TNF-α) in the striatum of PD mice (12). Germ-free mice failed to exhibit αSyn-mediated microglial activation and motor impairments during PD, suggesting the essential role of gut flora in driving PD pathophysiology (13). Yet, relatively little is understood about how the gut microbiota dysbiosis arises and the mechanisms by which specific commensal microbes play a role in PD etiology.

Notably, recent studies have proposed that one possible mechanism that microbes participate in PD etiology is through the regulation of microbe-derived metabolites in the circulation (13). Gut microbiota produces various catabolites involving fatty acids, secondary bile acids, and amino acids, which may modulate immune, metabolic, and neuronal responses of the host (14). Untargeted metabolomics is a global unbiased technology to obtain the metabolite profiles of biofluids, allowing us to extensively explore the metabolic alterations associated with PD initiation and progression (15). In fact, evidence is accumulating that intestinal microbiota contributes to the progression of other neurodegenerative diseases via altering metabolite levels in peripheral blood (16). However, only one clinical trial has characterized the disturbed metabolic profile in the serum of PD patients, and the in-depth mechanistic links between metabolites and PD pathogenesis are ambiguous (17). Besides, the association of gut microbiota community and serum untargeted metabolite profiles has not been established in a PD mice model yet, which hampers us from exploring the role of microbiota-related metabolism in PD pathogenesis. 1-Methyl-4-phenyl-1,2,3,6-tetrahydropyridine (MPTP) is a neurotoxic chemical that specifically damages DA neurons, which is commonly used to establish a PD mice model (18). Here we describe for the first time, to our knowledge, the landscape of serum untargeted metabolism of MPTP-induced PD mice. Meanwhile, we provided a detailed profile of a dysregulated microbiome-metabolome axis and evidenced that an MPTP-induced PD mice model could be used as an appropriate tool for basic research concerning the gut-brain axis of PD.

In this study, we demonstrated histopathological and functional deteriorations in the substantia nigra pars compacta-striatum system and colon in MPTP-induced PD mice. Next, by using 16S rRNA sequencing and ultrahigh-performance liquid chromatography coupled with Q Exactive HF-X mass spectrometry (UHPLC-QE/MS) untargeted metabolomics, the altered pattern of gut microbiome and serum metabolome was evaluated. Pearson’s correlation analysis showed that genera including *Lactobacillus*, *Adlercreutzia*, and metabolites concerning “taurine and hypotaurine metabolism” were identified as significantly correlated microbial groups or metabolic pathways with pathological and GI performance of PD. Furthermore, elevated level of bacteria-derived transporter and enzymes contributing to taurine (TAU) degradation was confirmed. More importantly, improvement of taurine metabolism is sufficient to prevent the motor dysfunction and DA neuron loss in the MPTP-treated mice. Collectively, our results uncover the profile of aberrant microbiota-metabolism links and shed light on the importance of microbe-mediated taurine metabolism in PD mice, which may offer unique insights into PD pathogenesis and therapeutic strategies.

## RESULTS

### MPTP treatment triggered motor impairment, DA neuron loss, and neuroinflammation in mice

**Fig 1:**
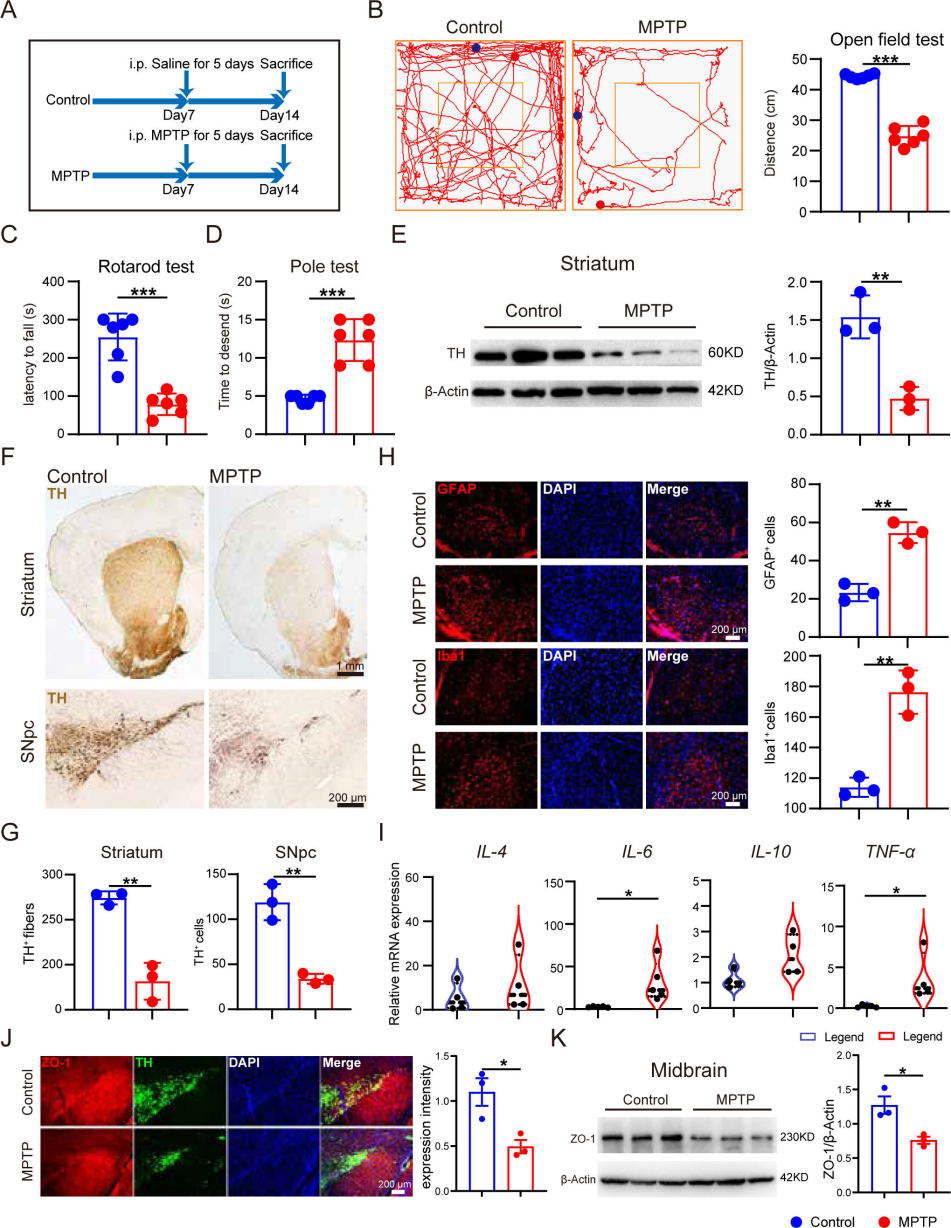
MPTP treatment triggered motor impairment, DA neuronal loss, neuroinflammation, and blood-brain barrier dysfunction in mice. Male C57BL/6 mice were domesticated for 7 days then were treated with 200-µL saline containing MPTP (30 mg/kg) or 200-µL saline via intraperitoneal (i.p.) injection every day for a total of five times, starting from day 8. On day 14, behavioral tests were performed to evaluate the motor function, and the mice were sacrificed to determine the pathology of PD by immunohistochemistry, immunofluorescence, and immunoblot. (**A**) The experimental flowchart. (**B**) Representative traces in the open field test, and quantification of performance in the open field test. Blue point: starting position; red point: ending position (*n* = 6). (**C and D**) Quantification of performance in the rotarod test and the pole test (*n* = 6). (**E**) Representative bands of tyrosine hydroxylase (TH) protein in striatum of one hemisphere determined by Western blotting (WB) and quantification of TH expression (*n* = 3). (**F and G**) Representative immunohistochemistry images and quantification of TH-positive fibers and neurons in striatum and substantia nigra pars compacta (SNpc) (*n* = 3). (**H**) Representative immunofluorescence images and quantification of Iba1-positive cells and GFAP-positive cells (*n* = 3). (**I**) Relative mRNA expression of pro-inflammatory cytokines in the striatum between control group and MPTP-treated group (*n* = 4–6). (**J**) Representative immunofluorescence images and quantification of zonula occludens-1 (ZO-1) intensity in SNpc (*n* = 3). (**K**) Representative bands of ZO-1 protein in the midbrain of one hemisphere determined by WB and quantification of ZO-1 expression (*n* = 3). Data are expressed as mean ± SD, and representative results are one of three independent experiments. All statistical differences were tested using unpaired two-tailed Student’s *t*-test. Quantification of TH-positive fibers and neurons, Iba1-positive cells, GFAP-positive cells, and ZO-1 protein was performed by ImageJ. *P < 0.05, **P < 0.01, ***P < 0.001. Abbreviations: DAPI, 4′,6-diamidino-2-phenylindole; GFAP, glial fibrillary acidic protein.

The flowchart of the experiment is shown in Fig. 1A. After MPTP injection for 5 consecutive days, three behavioral tests were performed to evaluate the motor function of mice: open test field test, rotarod test, and pole test. It was found that mice subjected to MPTP treatment exhibited reduced total distance traveled in the open field test when compared with mice in the control group (Fig. 1B). Meanwhile, MPTP-treated mice showed reduced latency in the rotarod test and required more time to descend through a pole (Fig. 1C and D). These results suggest that MPTP is sufficient for causing motor deficits in mice.

Considering that DA neuron loss is the typical pathological feature of PD, we next assessed alterations of DA neurons in the substantia nigra pars compacta (SNpc)-striatum system by determining the expression of tyrosine hydroxylase (TH). TH, a rate-limiting enzyme in the dopamine synthesis pathway, is widely recognized as a marker of the DA neurons (2, 19). A notable reduction in TH expression of striatum was observed in MPTP-treated mice by using Western blot (Fig. 1E). Consistently, TH expression in SNpc and the projecting neurofibril in the striatum of MPTP-treated mice was confirmed tremendously lower by utilizing immunohistochemistry (Fig. 1F and G). These results demonstrate the occurrence of DA neuronal loss in MPTP-treated mice.

**Fig 2:**
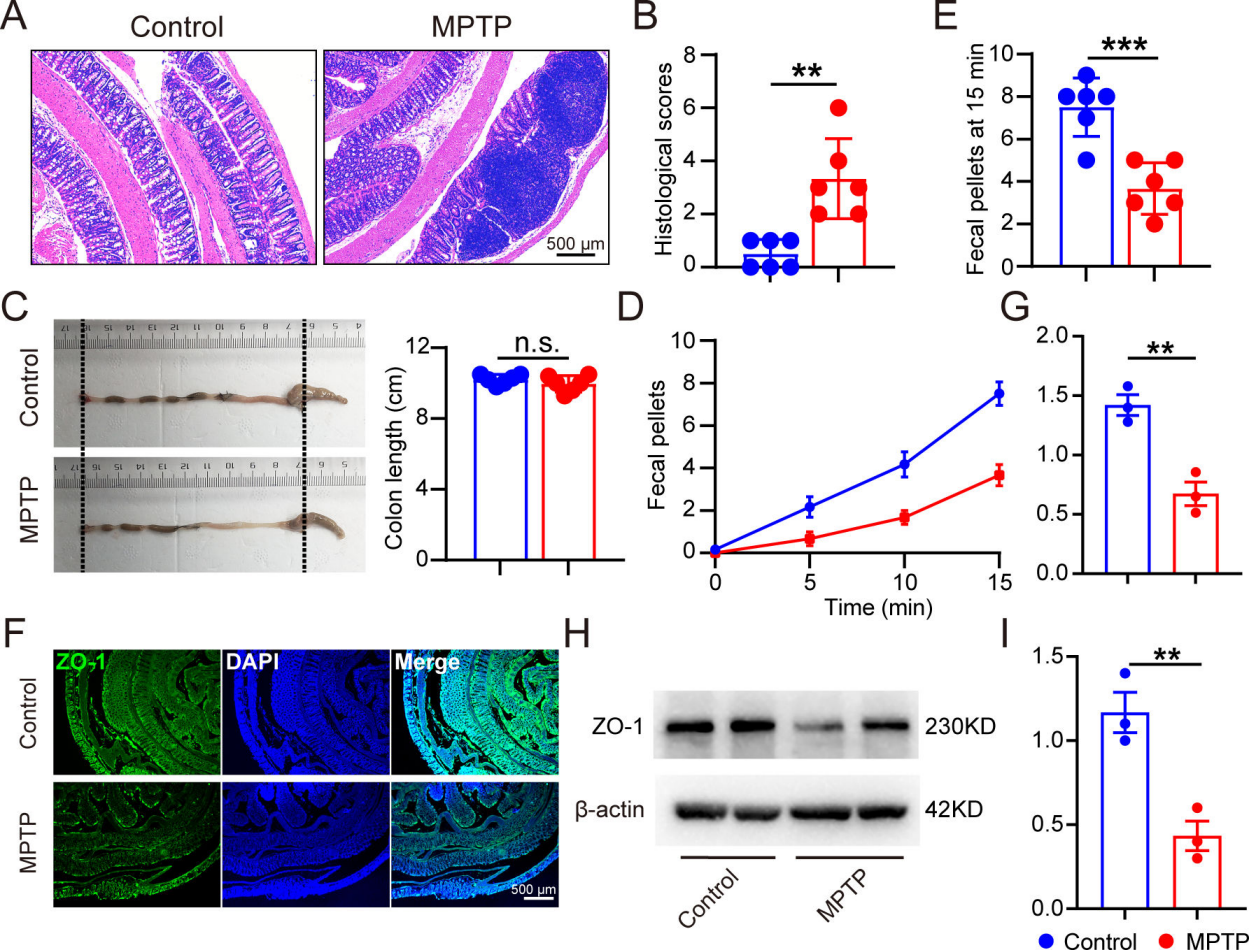
MPTP-treated mice exhibited colon histopathological and functional deterioration. Male C57BL/6 mice were domesticated for 7 days then were treated with 200-µL saline containing MPTP (30 mg/kg) or 200-µL saline via intraperitoneal injection every day for a total of five times, starting from day 8. On day 14, the mice were sacrificed to determine the histopathology of the intestine (*n* = 6). (**A and B**) Representative hematoxylin-eosin staining image of colon sections and corresponding histological scores. (**C**) Representative colon images and quantification of colon length. (**D**) Time course of fecal output in a novel environment over 15 min. (**E**) Total fecal pellets produced in 15 min. (**F and G**) Representative immunofluorescence images and quantification of ZO-1 intensity in the gut (*n* = 3). (**H and I**) Representative bands of ZO-1 protein in the intestine determined by WB and quantification of ZO-1 expression (*n* = 3). Data are expressed as mean ± SD, and representative results are one of three independent experiments. All statistical differences were tested using unpaired two-tailed Student’s *t*-test. **P < 0.01, ***P < 0.001. n.s., not significant.

It is widely recognized that the degeneration of DA cells in MPTP-induced mice is always accompanied by the activation of neuroglial cells (20). Then, we sought to evaluate microglial and astrocyte activation by determining the expression of their marker protein Iba1 and GFAP, respectively. Immunofluorescence of the SNpc demonstrated the significant elevation in the numbers of Iba1-positive cells and GFAP-positive cells after MPTP treatment (Fig. 1H). It was reported that glial cells are central to neuroinflammation of PD via modulation of cytokines, such as TNF-α, IL-4, IL-6, and IL-10 (20). Therefore, the levels of some neuroinflammatory mediators in the striatum of mice were detected using real-time quantitative PCR. The results revealed that mRNA levels of *IL-4*, *IL-6*, *IL-10*, and *TNF-ɑ* were elevated in MPTP-treated mice compared with controls (Fig. 2I), suggesting that MPTP treatment induced activation of glial cells and neuroinflammation.

To further explore the potential effect of MPTP on the blood-brain barrier (BBB), we assess the permeability of brain by determining the level of a tight junction protein, zonula occludens-1 (ZO-1). Immunofluorescence of Z0-1 in the SNpc showed a remarkable decline in the expression intensity after MPTP treatment (Fig. 1J). Consistently, a significant decrease in ZO-1 expression in the midbrain was observed in the MPTP mice using Western blot. These results suggest that MPTP treatment is responsible for the BBB disruption in mice.

Overall, these data demonstrate that MPTP-induced mice exhibit a comprehensive set of PD-relevant pathological changes.

### MPTP-treated mice exhibited colon histopathological and functional deterioration

As GI symptoms are recently regarded as a prodromal stage and critical hallmark of PD, we assessed histological and physiological alterations in the colon of MPTP-treated mice. The hematoxylin-eosin (H&E) staining sections displayed deleterious intestinal changes including infiltration of inflammatory cells and distortion of the epithelial architecture in PD mice (Fig. 2A). Although the colon length was unaffected (Fig. 2B), a marked decrease of total output of fecal pellets was confirmed in PD mice (Fig. 2C and D). Importantly, we discovered the decreased expression of ZO-1 protein in the intestine of MPTP-treated mice via immunofluorescence and immunoblotting, suggesting the damaged intestinal barrier function in the gut-brain axis of PD mice.

Altogether, we observed histopathological, functional, and barrier deteriorations in both the central nervous system and colon of MPTP-induced PD mice, suggesting that the colonic dysfunction may be implicated in PD etiology.

### Profile of gut microbiota was disturbed in MPTP-induced PD mice

**Fig 3:**
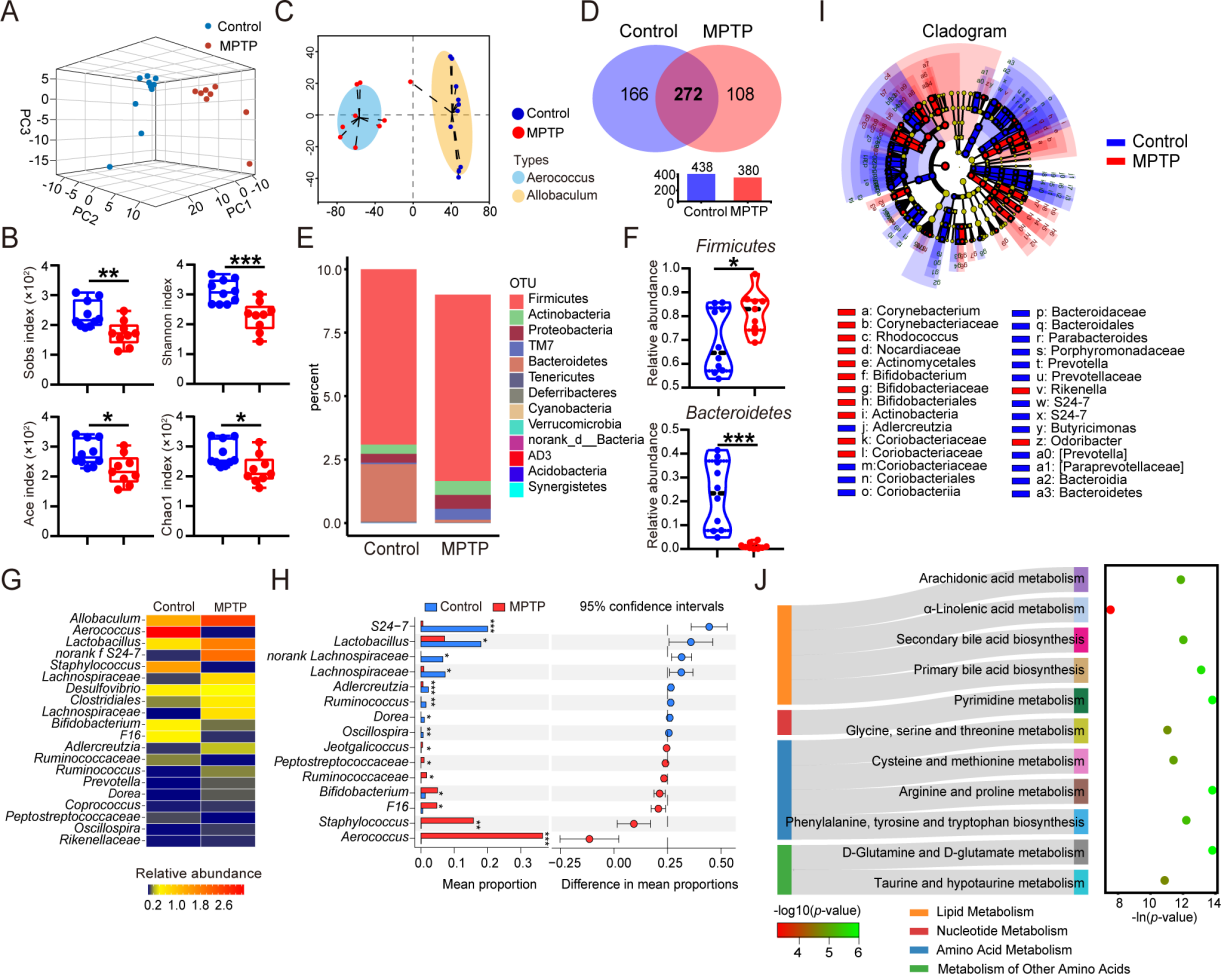
Profile of gut microbiota was disturbed in MPTP-induced PD mice. Male C57BL/6 mice were domesticated for 7 days then were treated with 200-µL saline containing MPTP (30 mg/kg) or 200-µL saline via intraperitoneal injection every day for a total of five times, starting from day 8. On day 14, fecal pellets were collected from the mice to detect the gut microbiota by using 16S rRNA sequencing (*n* = 9–10). (**A**) The 3D PCA plot of 19 samples from MPTP and control group. (**B**) The enterotype analysis at OTU level is shown. (**C**) Venn diagram of shared and unique OTUs. The histogram represents the number of OTUs of each group. (**D**) The α-diversity was estimated by Sobs, Shannon, Ace, and Chao1 indexes. (**E**) Composition of gut microbiota at phylum level. (**F**) Relative abundances of *Firmicutes* and *Bacteroidetes* are shown using a violin plot. (**G**) Relative abundances of top 20 genera are shown using a heatmap. (**H**) Statistical differences of top 20 genera between two groups were determined. (**I**) Cladogram of linear discriminant analysis scores for differentially abundant bacteria (phylum, class, order, and family). Only taxa meeting an linear discriminant analysis significant threshold of >2.0 are shown. (**J**) The Sankey diagram of significantly changed Kyoto Encyclopedia of Genes and Genomes hierarchical level 2 (left) and level 3 (right) classifications. Data are expressed as mean ± SD. Statistical differences were tested using unpaired two-tailed Student’s *t*-test in panels D and F and Welch’s *t*-test in panel H. *P < 0.05, **P < 0.01, ***P < 0.001.

Since numerous studies support that gut microbial groups serve as pivotal mediators in the gut-brain axis that is deeply involved in pathogenesis of PD, we assessed the gut microbiome by utilizing 16S rRNA sequencing across 19 fecal samples. The three-dimensional (3D) principal component analysis (PCA) plot (Fig. 3A) and phylogenic tree (Fig. 1A) showed that the structure of gut microbial composition in PD mice was remarkably distinct from that of control mice, and mice in the same group shared a high similarity. Subsequently, the bacterial community richness and diversity were determined. The downregulated Shannon index showed reduced diversity in PD mice, and the Sobs, Ace, and Chao1 index identified a descending richness after MPTP treatment (Fig. 3B). The enterotype classification is a novel method which was first reported in *Nature* by Bork et al. and is used to identify the dominant bacteria independent of age, gender, and nutritional status (21). The enterotype analysis revealed that *Aerococcus* was enriched in PD mice, while *Allobaculum* was enriched in control mice (Fig. 3C). Moreover, we examined the shared and unique bacterial operational taxonomic units (OTUs) among different groups. The shared OTUs from 19 samples are shown with a Venn diagram (Fig. 3D). These findings indicate the obvious difference in microbial community between PD mice and normal control.

To obtain in-depth knowledge of alterations in microbiota community structures of PD mice, taxonomic analysis at different levels was performed in all samples. The changes in microbial composition at phylum level are shown in Fig. 3E. It should be noted that the remarkably elevated abundance of *Firmicutes* and the reduced abundance of *Bacteroidetes* were observed in PD mice (Fig. 3F). Taxonomic analysis at genus level was performed, and the relative abundances of the top 20 genera were displayed using a heatmap (Fig. 3G). Notably, PD mice exhibited significant increment of *Aerococcus*, *Staphylococcus*, *Bifidobacterium*, and *Jeotgalicoccus*, whereas the *norank S24−7*, *Lactobacillus*, *Lachnospiraceae*, *Adlercreutzia*, and *Dorea* showed the opposite change (Fig. 3H). The constitution and statistical analysis of taxa at class, order, and family levels were also displayed (Fig. 1B through G). The mean proportions and statistical results of all flora at different levels are provided in Table S1.

To further delineate the core bacterial group in PD mice, linear discriminant analysis coupled with effect size (LEfSe) analysis was performed. The results revealed that *Aerococcus* was enriched in the PD mice, while *Lactobacillus* was enriched in the controls [linear discriminant analysis (LDA) score >2.0, *P* < 0.05; Fig. 3I; Table S2]. Furthermore, LDA analysis showed that *Aerococcus* and *Staphylococcus* were identified as core genera in PD mice, whereas *norank S24−*7 and *Lactobacillus* were marked as predominant groups in the control group (LDA score >4.0, *P* < 0.05; Fig. 1G).

To interpret the functions of these crucial genera, we analyzed the Kyoto Encyclopedia of Genes and Genomes (KEGG) pathway based on the phylogenetic investigation of communities by reconstruction of unobserved states 2 (PICRUST2) method. The differentially enriched KEGG pathway level 2 included “lipid metabolism,” “nucleotide metabolism,” “amino acid metabolism,” and “metabolism of other amino acids.” The corresponding pathway level 3 included taurine and hypotaurine metabolism; “α-linolenic acid metabolism”; “glycine, serine, and threonine metabolism”; “cysteine and methionine metabolism”; “D-glutamine and D-glutamate metabolism”; “phenylalanine, tyrosine, and tryptophan biosynthesis”; “arginine and proline metabolism”; “pyrimidine metabolism”; “primary bile acid biosynthesis”; “secondary bile acid biosynthesis”; and “arachidonic acid metabolism” (Fig. 3J). Together, our results demonstrated a remarkable shift in gut flora profile and microbial function in MPTP-induced PD mice.

### Serum metabolomic profiling reveals an apparent disparity between MPTP-induced PD mice and control mice

**Fig 4:**
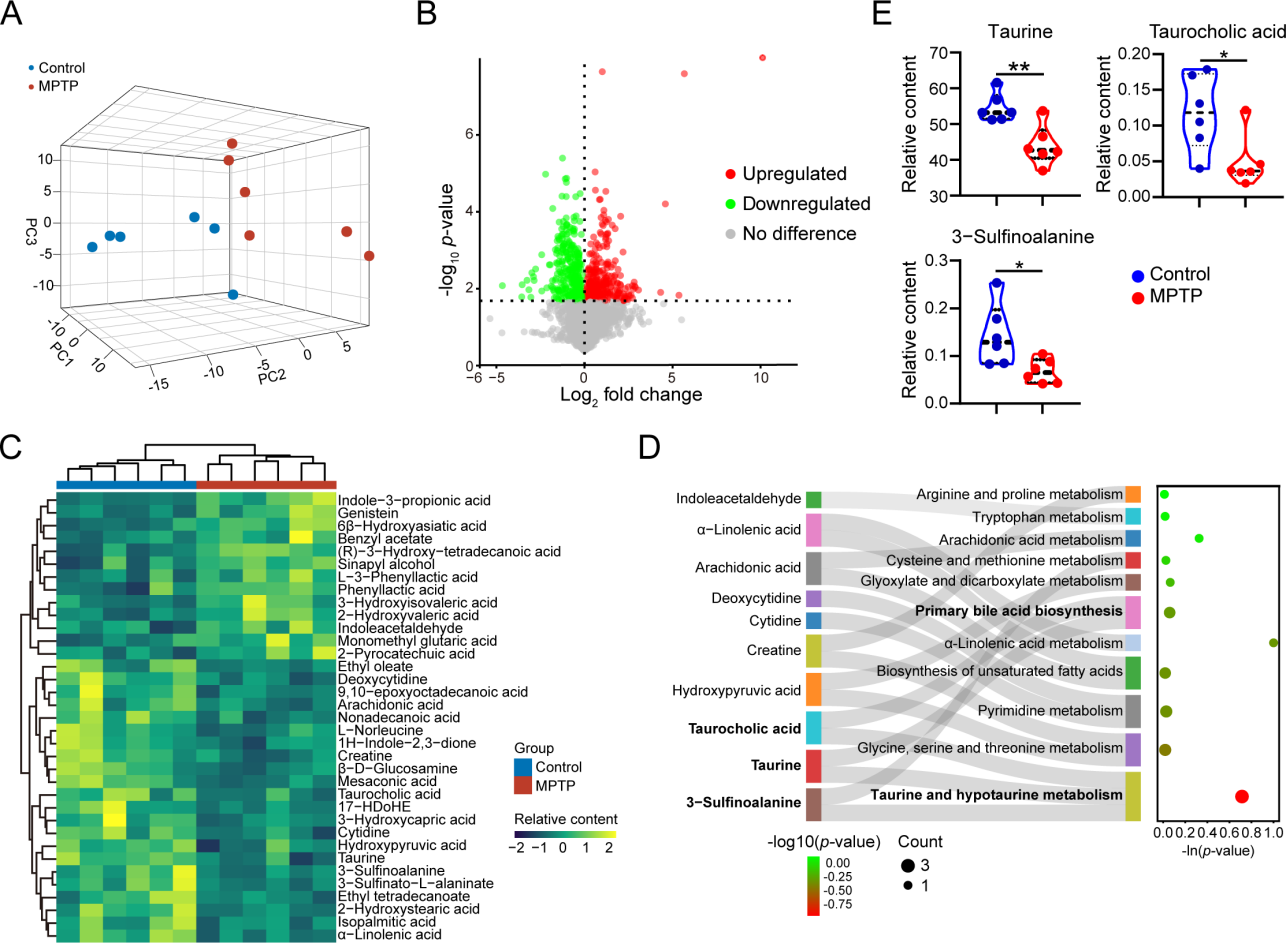
Serum metabolomic profiling reveals an apparent disparity between MPTP-treated mice and control mice. Male C57BL/6 mice were domesticated for 7 days then were treated with 200-µL saline containing MPTP (30 mg/kg) or 200-µL saline via intraperitoneal injection every day for a total of five times, starting from day 8. On day 14, the serum samples were collected from the mice to detect the untargeted metabolomics by UHPLC-QE/MS (*n* = 6). (**A**) The 3D PCA plot of 12 samples from MPTP and control group. (**B**) Volcano plot of the differential metabolites between the MPTP-treated group and the control group (VIP value >1 and *P* value < 0.05). Each dot represents a detected metabolite. (**C**) Relative contents of 35 annotated differential metabolites are shown using a heatmap. (**D**) Sankey diagram of mapped pathways and correspondingly differential metabolites. (**E**) Relative contents of differentially changed metabolites involving taurine metabolism pathway using a violin plot. Data are expressed as mean ± SD. Statistical differences were tested using unpaired two-tailed Student’s *t*-test in E. *P < 0.05, **P < 0.01.

Since metabolites are recognized as crucial communicators of the host-microbiota axis, we next explored the serum metabolic profiles of the two groups. A total of 4374 metabolites were detected and quantified by UHPLC-QE/MS analysis. The 3D PCA plot showed a significant difference in the clustering of metabolite distribution between PD mice and controls (Fig. 4A). After thresholding metabolites with a variable importance of projection (VIP) value of >1 and a *P* value of <0.05, we filtered 565 metabolites that significantly differed in content between MPTP-treated mice and control mice (Fig. 4B). A total of 35 differential metabolites were annotated, and the levels of these metabolites were summarized in a hierarchical cluster analysis (Fig. 4C; Table S3). The KEGG pathway analysis was performed to investigate the signaling pathways that were impacted by the differential metabolites. Eleven pathways were identified, including taurine and hypotaurine metabolism; glycine, serine, and threonine metabolism; pyrimidine metabolism; biosynthesis of unsaturated fatty acids; α-linolenic acid metabolism; primary bile acid biosynthesis; glyoxylate and dicarboxylate metabolism; cysteine and methionine metabolism; arachidonic acid metabolism; tryptophan metabolism; and arginine and proline metabolism (Fig. 4D). Among them, the taurine and hypotaurine metabolism pathway showed the highest impact value (impact value = 0.71, *P* < 0.001; Table S4), and the differentially altered metabolites involved in this pathway are taurine, 3-sulfinoalanine (also known as cysteine sulfinic acid), and taurocholic acid (Fig. 4E). In summary, serum metabolome alterations in PD mice are characterized by the taurine metabolic disorder.

### Correlations between changes in gut microbiota profile, serum metabolism alterations, and PD-related pathological results

**Fig 5:**
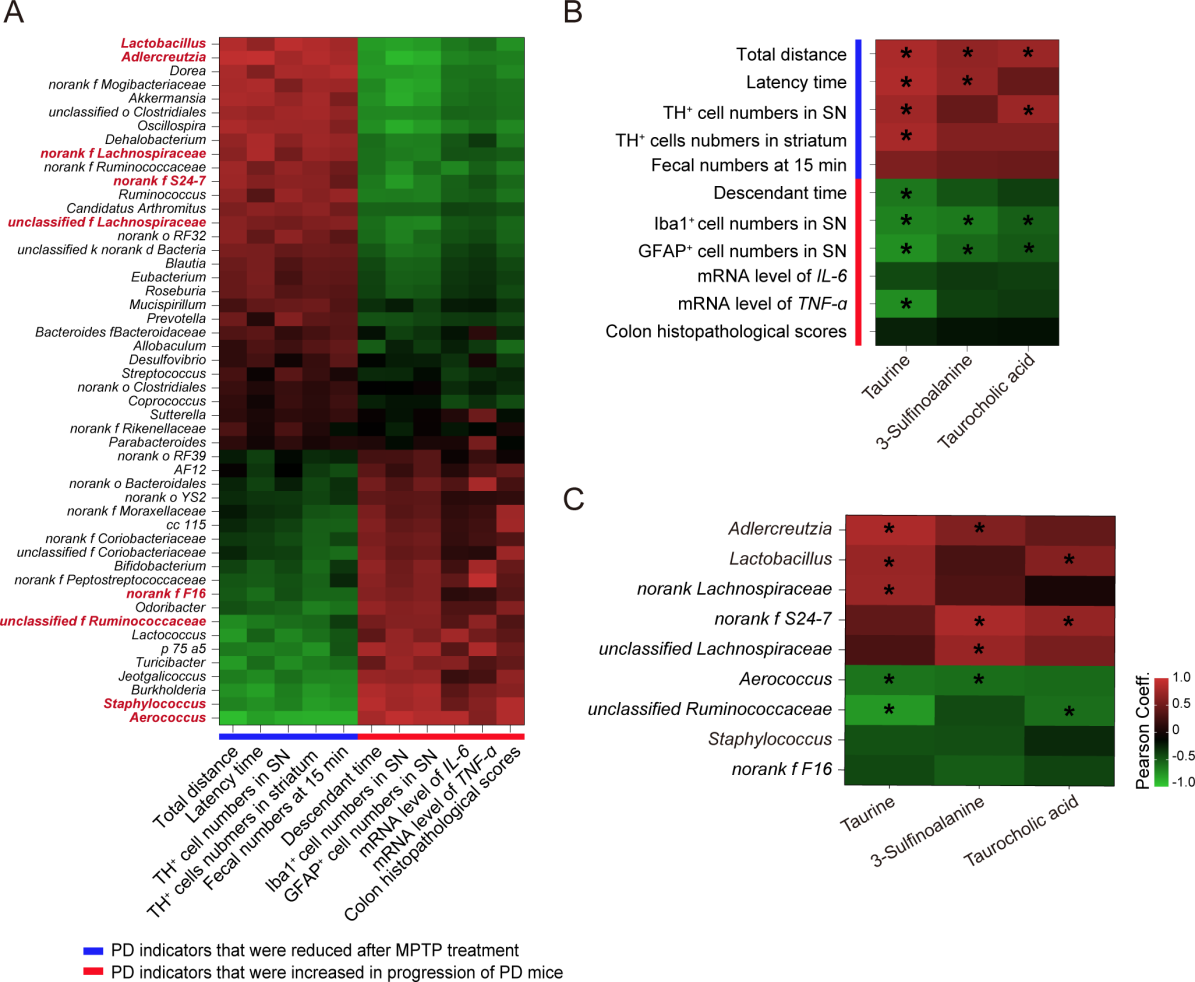
Correlations between changes in gut microbiota profile, serum metabolism alterations, and PD-related pathological results. (**A**) Heatmap of Pearson’s correlation coefficients between the abundances of top 50 genera and PD-associated results. Bacterial groups in red represent the top 10 altered genera. (**B**) Heatmap of Pearson’s correlation coefficients between PD-associated results and levels of differential metabolites in taurine metabolism pathway. (**C**) Heatmap of Pearson’s correlation coefficients between the abundances of vital gut flora and taurine metabolism-associated metabolites. Vital gut flora was identified as top 10 altered genera with Pearson’s correlation coefficient of >0.5. Pearson’s correlation coefficients are shown by color. Red indicates a negative correlation, and the blue indicates a positive correlation between two variables. *P < 0.05.

To better interpret the role of the microbiota-metabolite axis in PD pathology, correlation analysis was performed among microbiota community, microbial metabolomics, and PD associated features. Genera of top 50 in abundance were selected for this analysis. It is worth noting that *Lactobacillus*, *Adlercreutzia*, and *norank Lachnospiraceae*, *unclassified Lachnospiraceae*, and *norank S24-7* were positively correlated with PD indicators that were reduced after MPTP treatment and negatively correlated with those increased during progression of PD. Meanwhile, *Aerococcus*, *Staphylococcus*, *unclassified Ruminococcaceae*, and *norank F16* showed the opposite results (Fig. 5A; Table S5). The correlation analysis at the OTU level is shown in Table S6. These data suggest the potential neuroprotective role of *Lactobacillus*, *Adlercreutzia*, *norank Lachnospiraceae*, *unclassified Lachnospiraceae*, and *norank S24-7* in PD pathology. Conversely, high abundances of *Aerococcus*, *Staphylococcus*, *unclassified Ruminococcaceae*, and *norank F16* may be contributors to the progression of PD.

Subsequently, a correlation analysis between three selected metabolites concerning the taurine and hypotaurine metabolism pathway and PD-associated features was performed. The levels of taurine, 3-sulfinoalanine, and taurocholic acid were positively correlated with PD indicators that were reduced in response to MPTP treatment and negatively correlated to those increased in PD mice (Fig. 5B). As expected, the expression of taurine showed a significant association with almost all PD indicators, except the colon feature named “the fecal numbers at 15 min.” These findings imply that metabolites including taurine, 3-sulfinoalanine, and taurocholic acid may exert beneficial effects on PD progression, among which taurine is probably the key mediator.

To determine the possible linkage between gut microbiota and taurine metabolism, Pearson’s correlation between PD-associated genera and differential metabolites involving taurine metabolism was performed. Genera that were closely correlated with any of selected three metabolites included *Aerococcus*, *Lactobacillus*, *Adlercreutzia*, *unclassified Ruminococcaceae*, *norank Lachnospiraceae*, *unclassified Lachnospiraceae*, and *norank S24-7* (Fig. 5C), implying their important part in the modulation of taurine metabolism. Most of all, the abundances of *Aerococcus*, *Lactobacillus*, *norank Lachnospiraceae*, and *unclassified Ruminococcaceae* were remarkably correlated with the expression level of taurine (Fig. 5C). Taken together, our data support the notion that dysregulation of the microbiota-serum metabolites axis may be involve in the etiology of PD.

### The disturbed metabolism of taurine is possibly attributed to upregulated microbial taurine degradation in PD mice

**Fig 6:**
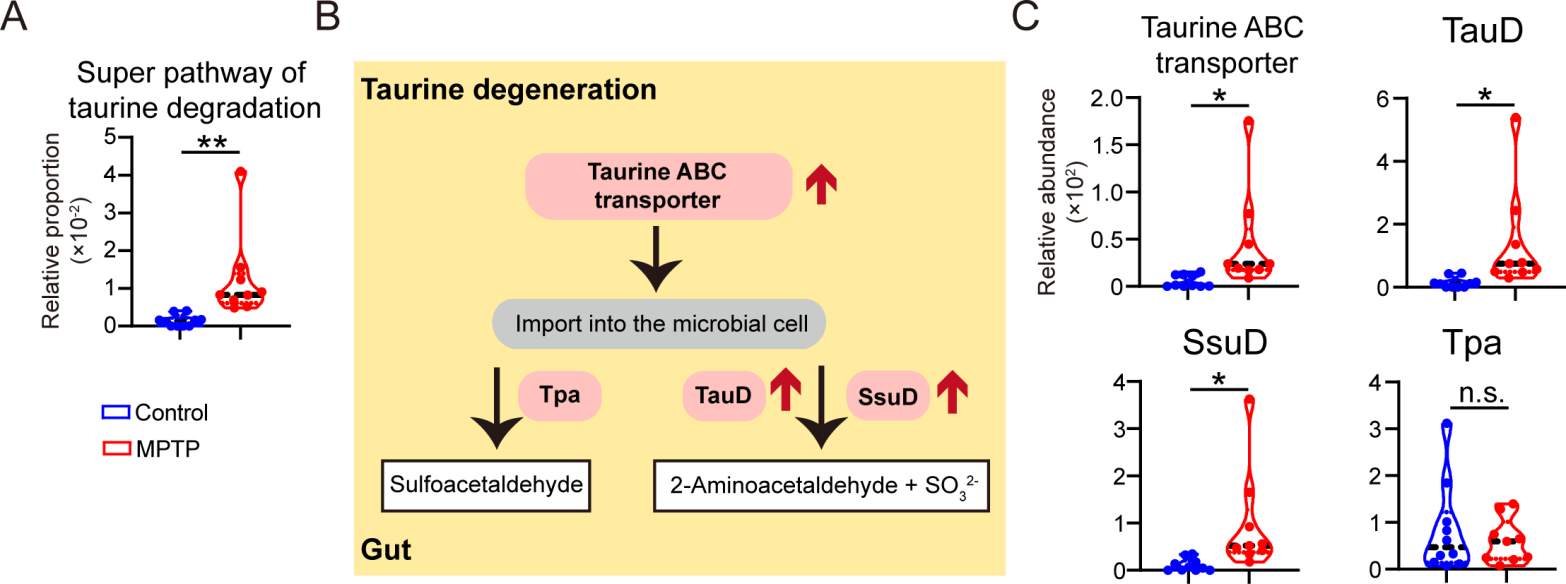
The disturbed metabolism of taurine is possibly attributed to upregulated microbial taurine degradation in PD mice. (**A**) Relative proportion of super pathway of taurine degradation (MetaCyc pathway PWY-1541). (**B**) The schematic diagram of taurine degradation. (**C**) Relative abundances of taurine ABC transporter, TauD, SsuD, and Tpa between PD mice and controls. Data are expressed as mean ± SD. Statistical differences were tested using unpaired two-tailed Student’s *t*-test in panels A and C. *P < 0.05, **P < 0.01. n.s., not significant. Abbreviations: SsuD, alkanesulfonate dioxygenase; TauD, taurine dioxygenase; Tpa, tau-pyruvate aminotransferase.

To explore the potential mechanisms by which gut microbiota modulates taurine metabolism, we further analyzed the KEGG enzyme and MetaCyc metabolic pathway in microbes (22). Importantly, PD mice showed a remarkable enrichment of super pathway of taurine degradation (Fig. 6A), indicating that taurine degradation may be the crucial process contributing to taurine metabolism. The diagram of microbial taurine degeneration in PD mice is displayed in Fig. 6B. Taurine is imported into the bacteria via taurine ABC transporter and is ultimately converted to sulfite via taurine dioxygenase (TauD) or alkanesulfonate dioxygenase (SsuD) or tau-pyruvate aminotransferase (Tpa) (23). Notably, the predicted abundances of taurine ABC transporter, TauD, and SsuD were evidently upregulated in PD mice, while the expression of Tpa is unaltered (Fig. 6C). In this part, we conclude that altered microbiota community may disturb taurine degeneration via regulating the microbial expressions of taurine ABC transporter, TauD, and SsuD, which consequently contribute to the etiology of PD.

### Taurine supplementation alleviates behavioral deficits, DA neuronal loss, and microglial activation in MPTP-treated mice

**Fig 7:**
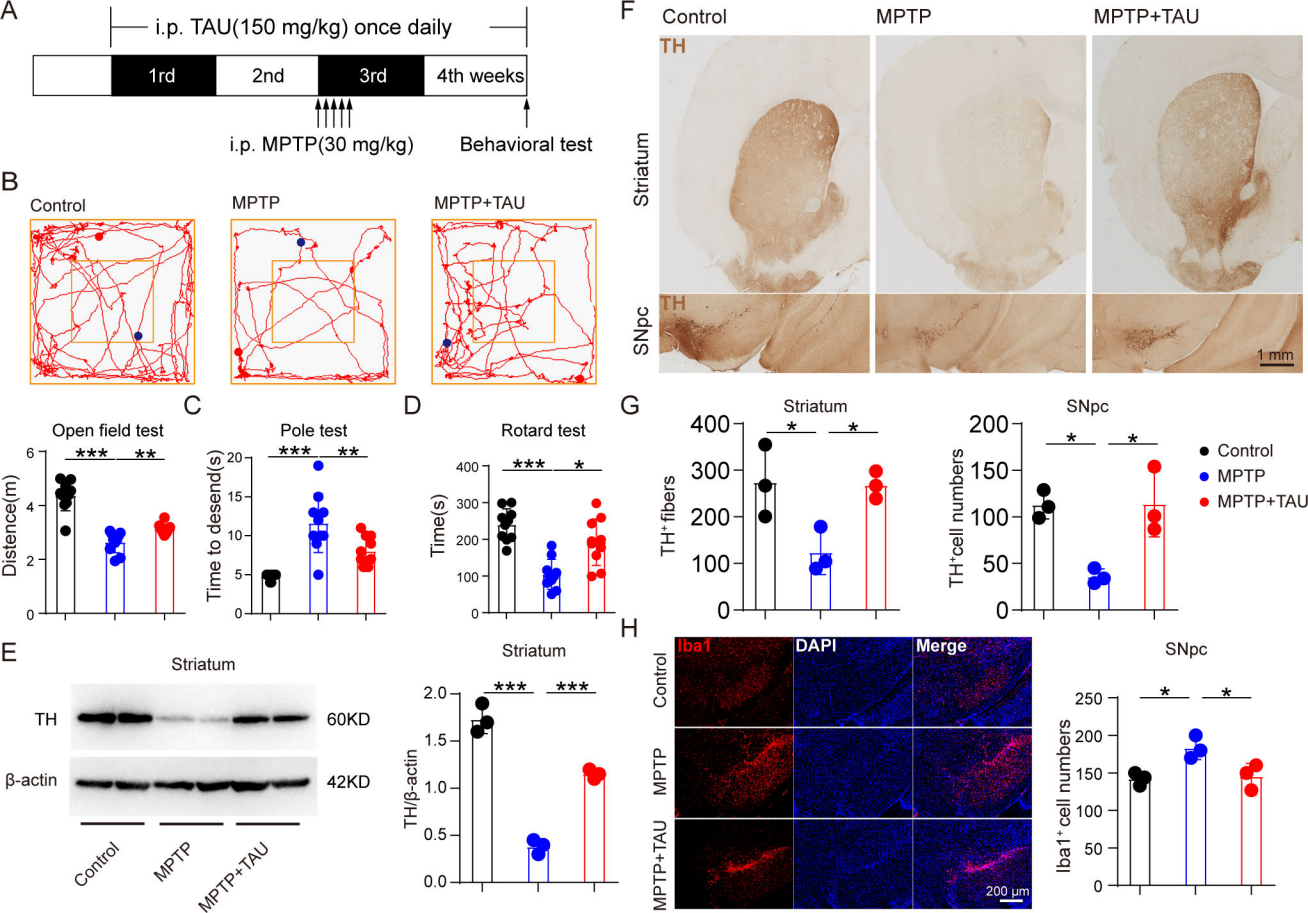
Taurine administration prevented motor deficits, DA neuronal loss, and microglial activation in MPTP-treated mice. Male C57BL/6 mice were domesticated for 7 days then were treated with 200-µL saline containing taurine (150 mg/kg) or 200-µL saline via intraperitoneal injection every day for 4 consecutive weeks. Two hundred-microliter saline containing MPTP (30 mg/kg) or 200-µL saline via intraperitoneal injection every day for a total of five times, starting from day 15. On day 28, behavioral tests were performed to evaluate the motor function, and the mice were sacrificed to determine the pathology of mice by immunohistochemistry, immunofluorescence, and immunoblot. (**A**) The experimental flowchart. (**B**) Representative traces in the open field test and quantification of performance in the open field test. Blue points: starting positions; red points: ending positions (*n* = 10). (**C and D**) Quantification of performance in the pole test and the rotarod test (*n* = 10). (**E**) Representative bands of TH protein in the striatum of one hemisphere determined by WB and quantification of TH expression (*n* = 3). (**F and G**) Representative immunohistochemistry images and quantification of TH-positive fibers and neurons in striatum and SNpc (*n* = 3). (**H**) Representative immunofluorescence images and quantification of Iba1-positive cells (*n* = 3). Data are expressed as mean ± SD, and representative results are one of three independent experiments. All statistical differences were tested using one-way analysis of variance. Quantification of TH-positive fibers and neurons and Iba1-positive cells was performed by ImageJ. *P < 0.05, **P < 0.01, ***P < 0.001.

To explicit the direct causal relationship between taurine metabolism and pathology of PD, MPTP-treated mice were given TAU supplementation through intraperitoneal injection for 4 weeks (Fig. 7A). On the last day of the treatment, three behavioral tests were measured to assess the motor ability of mice. It was found that MPTP mice that received TAU treatment traveled a longer distance in the open field test when compared with the MPTP group (Fig. 7B). Consistently, MPTP + TAU-treated mice showed increased latency in the rotarod test and spent fewer time descending through a pole (Fig. 7C and D). These data suggest that taurine supplement relieves motor deficits of MPTP-treated mice. To further evaluate the effect of taurine treatment on DA neuron loss, we observed alterations of DA neurons in the SNpc-striatum system. Expectedly, a significant increase in TH expression of the striatum was found in the MPTP + TAU group compared with mice treated with MPTP (Fig. 7E). Consistently, TH-positive neurons and neurofibrils were notably elevated in MPTP + TAU-treated mice compared with MPTP-treated mice (Fig. 1F and G). Besides, taurine treatment reduced the numbers of Iba1-positive cells in the SNpc. These results demonstrate that elevated taurine metabolism is sufficient for preventing motor impairment, DA neuronal loss, and microglial activation in MPTP-treated mice.

## DISCUSSION

**Fig 8:**
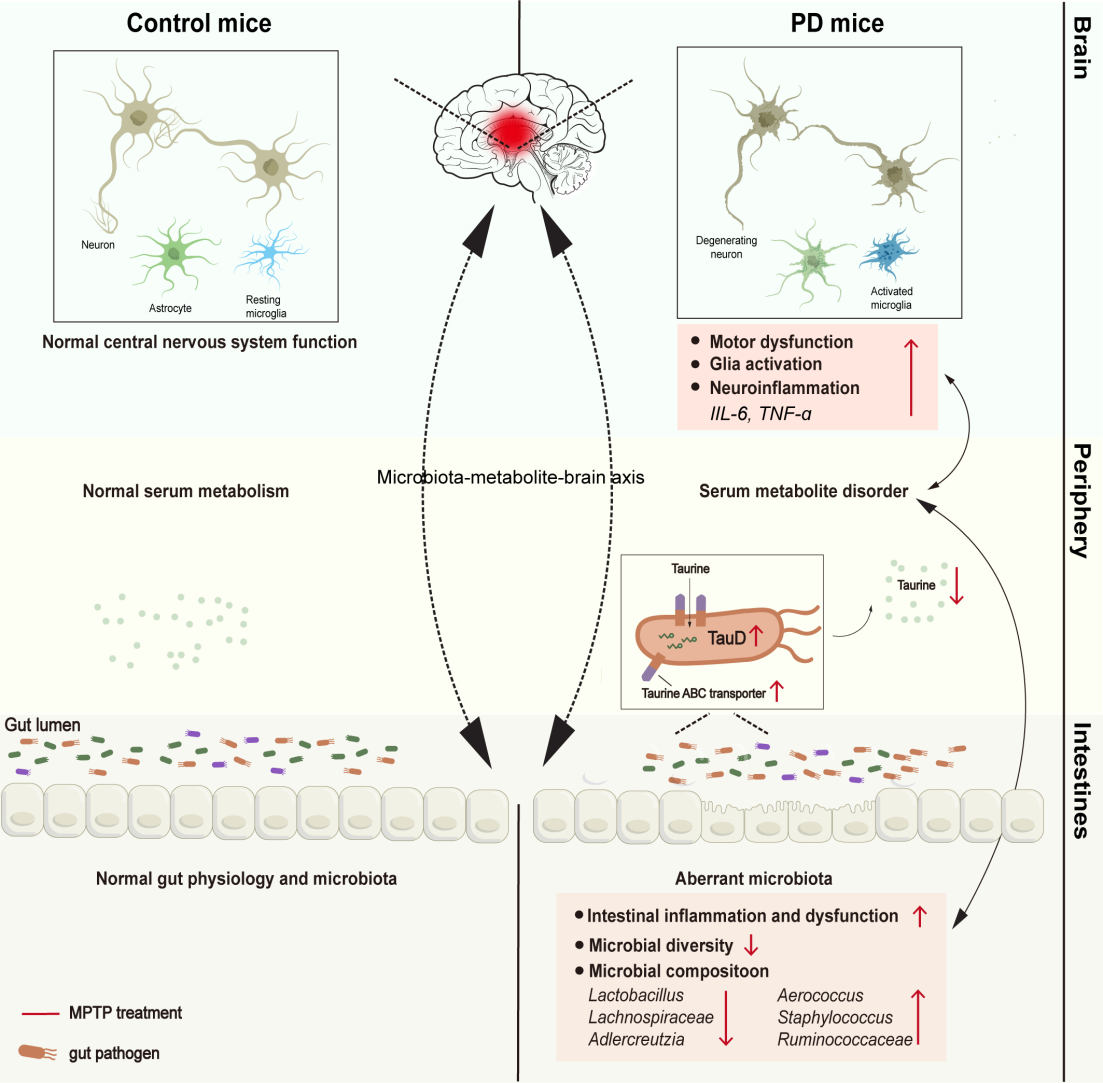
The schematic diagram of aberrant gut microbiota-taurine metabolite axis in PD mice.

In this study, we observed marked taxonomic differences in PD mice, including an enlarged abundance of *Aerococcus* and decreased abundances of *Lactobacillus* and *Adlercreutzia*. Meanwhile, altered levels of metabolites concerning taurine and hypotaurine metabolism were identified. Furthermore, altered genera such as *Lactobacillus* and *Adlercreutzia*, and differential metabolites of taurine and hypotaurine metabolism such as taurine were closely correlated with pathological and GI performance of PD (Fig. 8). Upregulated expression of bacterial enzymes involving taurine degradation was confirmed. More importantly, taurine supplementation alleviates behavioral deficits, DA neuronal loss, and microglial activation in MPTP-treated mice. Taken together, our data provide evidence for a taurine-based microbiota-metabolism axis that is closely associated with PD severity, highlighting the important role of restoring the gut microbial ecosystem in PD treatment.

Microbial metabolites are widely accepted as vital mediators in microbiota-involved regulation of PD initiation and progression (24). Taurine is recognized as the key metabolite of the taurine and hypotaurine metabolism pathway, which is markedly reduced in PD mice and evidently associated with the motor function and pathology in our study. As a sulfur-containing amino acid, taurine is found highly enriched in the substantia nigra (SN) and striatum and is reported to play an essential role in the etiology of PD via modulating neuroinflammation, oxidative stress, and αSyn pathogenesis (25–27). Recent studies also show that the plasma taurine level is sharply decreased in PD patients and negatively correlated with motor severity, suggesting a neuroprotective role of taurine in PD (28, 29). Despite these findings, the mechanism which contributes to the lower level of taurine remains obscure. A recent study reported that taurine is unable to be further metabolized by the host, and bacteria play an important role in taurine metabolism (30); we hypothesized that the microbial taurine catabolism is a crucial process causing reduced levels of taurine in PD mice. Indeed, it was found that abundances of *Lactobacillus*, *Adlercreutzia*, *Aerococcus, norank Lachnospiraceae*, and *unclassified Ruminococcaceae* are notably correlated with the level of taurine, and expression of enzymes responsible for taurine degradation was determined to be upregulated in PD mice. Thus, our results indicate that these five genera may impact PD development through modulating taurine metabolism.

*Lactobacillus* species have long been accepted as an important component of probiotics that exerts beneficial effects on gut barrier integrity and microbial community health (31). Recent studies also show that *Lactobacillus* treatment can alleviate PD progression via reducing microglial reactivity and regulating oxidative damage (32–34). Our data demonstrated that the abundance of *Lactobacillus* was positively related to serum taurine level*,* implying that *Lactobacillus* might be involved in taurine metabolism in PD mice. In agreement with our findings, administration of *Lactobacillus* is reported to notably increase taurine level and therefore ameliorate inflammation in high-fat diet-treated mice (35). Similarly, *Lactobacillus* supplementation can maintain high levels of taurine by reducing taurine excretion via the urine in horse (36). These findings support our notion that *Lactobacillus* deficiency can drive reduction of taurine level and impair the protective effect of taurine in PD. From a therapeutic perspective, strategies that aim to enlarge the abundance of *Lactobacillus* such as probiotic treatment may contribute to restoring the content of taurine and therefore provide benefit for PD patients.

*Adlercreutzia* is previously reported as an equol-producing genus in human feces (37). We found that PD mice showed a significant contraction of *Adlercreutzia*, and the abundance of *Adlercreutzia* was negatively correlated with microglial cell numbers in SNpc and the striatum, suggesting *Adlercreutzia* might display an antineuroinflammatory effect on PD mice. Consistent with our findings, Subedi et al. have documented that *Adlercreutzia* exhibited strong antioxidant and anti-inflammatory properties via generating equol, which was found to mitigate microglia-involved neuroinflammation through inhibiting NF-κB activation (38). Notably, we here emphasize the positive correlation between *Adlercreutzia* and taurine, which provide novel evidence that taurine may be a critical metabolite responsible for benefit of *Adlercreutzia* in PD.

It is found that *Lachnospiraceae* can alleviate inflammatory reactions by promoting microglial apoptosis in PD (39). The abundance of *Lachnospiraceae* is elevated in PD patients with GI complications and is significantly related to the disease severity (40–44). Our study found a positive relationship between the abundance of *Lachnospiraceae* and taurine level. Interestingly, several studies have reported that the abundance of *Lachnospiraceae* was increased after taurine treatment, perhaps since that taurine could be used as substrate for bacterial growth in the colon (45, 46). Our findings raise the possibility that taurine can enlarge the abundance of *Lachnospiraceae* and exert its protective effect on PD pathology.

*Aerococcus* and *Ruminococcaceae* are the groups both identified to be negatively correlated with levels of taurine in the serum. *Aerococcus* is increasingly recognized as a human pathogenic bacterium in urinary tract infection and infective endocarditis (47, 48). However, *Aerococcus* has not been reported in previous research of PD. *Ruminococcaceae* is a group of anaerobic bacteria that is colonized in the colonic mucosa of humans (49). Consistent with our results, several studies reported that the abundance of *Ruminococcaceae* was significantly reduced in PD patients (50, 51). Given that *Aerococcus* and *Ruminococcaceae* are confirmed incremented and strongly related to pathological changes, research is needed to deeply examine their roles in PD. In addition to the above microbial groups, several differential genera, including *norank S24-7* and *Staphylococcus*, were found markedly correlated with the motor function and pathology of PD mice. Consistently, the family *S24-7*, also known as *Muribaculaceae*, was reported to decrease in PD, while *Staphylococcus* was found elevated (52, 53), suggesting that *S24-7* and *Staphylococcus* are probably involved in the pathology of PD. Besides, two differentially expressed metabolites, taurine and taurocholic acid (TCA), were found to involve the primary bile acid biosynthesis pathway, which was also disturbed in PD mice. TCA, recognized as a primary BA, is formed by taurine conjugating with cholic acid and is reported to be essential for solubilization and transport of lipid (54). Our data revealed that reduced level of TCA is notably correlated with the motor function and pathological results of PD mice, indicating that TCA might exert a beneficial effect on PD progression. However, research which explores the relationship between TCA and PD pathology is not found, suggesting that more studies are required to examine the role of TCA in the initiation and progression of PD. Together, these findings will require assessment and validation in future studies.

In this context, our work highlights the key contribution of the taurine-based microbiome-metabolism axis to MPTP-induced pathology. *Lactobacillus* and *Adlercreutzia* were identified as the most correlated genera that participate in taurine metabolism. It should be noted that crucial microbial mediators may be represented as potential biomarkers essential to the progression of PD. Most of all, novel therapeutic approaches based on modulation on these crucial gut microbiota or taurine metabolism may exert a desirable effect on the GI disturbances complementary to the current PD treatment.

## MATERIALS AND METHODS

### Animals and treatments

Male C57BL/6 mice (8–10 weeks, 25–30 g) from Shanghai SLAC Laboratory Animal Co. Ltd were housed in a specific pathogen-free environment (22.5 ± 5.0°C, 43 ± 5% humidity, and 12/12-h light/dark).

Thirty milligrams per kilogram MPTP (Cat# M0896; Sigma-Aldrich, St Louis, MO, USA), prepared using sterile saline, was injected intraperitoneally once daily for 5 consecutive days to establish the PD model, whereas controls were injected with sterile saline solution (0.9% NaCl, wt/vol). Mice were sacrificed 2 days after the last injection.

For the taurine supplementation experiment, 150-mg/kg taurine (Cat# T0625, Sigma-Aldrich), prepared using sterile saline, was injected intraperitoneally every day for 4 weeks (55). Two weeks after the taurine administration, MPTPs were given to construct the PD mice model.

### Behavioral tests

#### Open field test

Open field test was conducted to evaluate the spontaneous locomotor activity of mice. Before the test, the mice were allowed to be acclimatized to the experimental environment for half an hour. The mice were permitted to investigate freely in the chamber for 5 min, and all the activities were recorded through an automated video system (MED Associates, Georgia, VT, USA). The total distances were calculated to assess the motor function.

#### Rotarod test

The rotarod test is used to assess the coordination and motor learning of mice. Mice were placed in five separate chambers on a spinning rotarod which can accelerate from 4 to 40 rpm within 5 min (Ugo Basile, Comerio, Italy). The latency to fall from the rotarod was recorded. All mice are allowed to train for 2 weeks before the final tests. Three trials were repeated, and the average latency time was used for analysis.

#### Pole test

The pole test was performed to estimate the motor coordination of mice. Mice were allowed to climb down the wooden pole, and the time spent from releasing the mice on the top latency to the one hind limb reaching the base was recorded. All mice are required to train three times daily for 5 consecutive days before the final tests. Three trials were performed, and the average time was measured.

### Western blotting

Brain tissues were lysed using RIPA buffer (Sigma-Aldrich) and centrifuged at 12,000 *g* and 4°C for 20 min to obtain the total protein in the supernatant. The protein concentrations were estimated by the bicinchoninic acid Protein Assay Kit (Thermo Fisher Scientific, Rockford, IL, USA). Then the protein was boiled in an SDS-loading buffer for 10 min to denature. The appropriate amount of protein was run on a 10% sodium dodecyl sulfate-polyacrylamide gel electrophoresis, transferred to 0.22-μm polyvinylidene fluoride membranes, and the membranes were blocked with 5% fat-free milk at room temperature for 1 h. The membranes were incubated at 4°C overnight with primary antibodies and were incubated at room temperature for 1 h with the horseradish peroxidase (HRP)-conjugated secondary antibody. Finally, enhanced chemiluminescence reagent (Thermo Fisher Scientific) was used to develop the blots, and band density was calculated using ImageJ (v.1.49). The following antibodies were used: anti-TH antibody (1:1,000, Cat# ab112; Abcam, Cambridge, MA, USA), antiβ-actin antibody (1:1,000, Cat# 8457S; Cell Signaling Technology, Berkeley, CA, USA), anti-ZO-1 antibody (1:1,000, Cat# 21773–1-AP; Proteintech, China), and goat anti-rabbit antibody (1:10,000, Cat# ab6721; Abcam).

### Immunohistochemistry and immunofluorescence

For immunohistochemistry, the brains were separated in paraformaldehyde (PFA) solution at 4°C overnight and embedded with 3% agarose in phosphate-buffered saline. Coronal sections were gathered using Vibratomes (Leica Biosystems, VT1000) at a thickness of 30 µm. Sections were transfrred into three-percent H_2_O_2_ solution for 10 min at room temperature following 10% donkey serum. For immunofluorescence, sections were blocked with 10% donkey serum for 30 min. Sections were incubated with the primary antibody anti-TH, anti-Iba-1, and anti-GFAP overnight at 4°C. Then the sections were incubated with an HRP-conjugated antibody and stained using DAB kit (DAB kit, Vector Laboratories). Fluorescein was combined with a fluorescent secondary antibody. Images were obtained using a fluorescent microscope (Leica, Wetzlar, Germany). The quantifications were conducted using ImageJ (v.1.49). The following antibodies were used: anti-Iba-1 antibody (Cat# ab5076, Abcam), anti-GFAP antibody (G3893, Sigma-Aldrich), goat anti-rabbit antibody (Cat# ab6721, Abcam), Donkey anti-mouse antibody (Cat# A21203, Invitrogen, Carlsbad, CA, USA), and donkey anti-goat antibody (Cat# A32758, Invitrogen).

### H&E staining and histological analysis of the intestine

Colon tissues were fixed with 4% PFA overnight and embedded with paraffin. Hematoxylin-eosin staining was performed according to the manufacturer’s instruction. Images were acquired using phase-contrast microscope (Leica). Histological scores obtained by a blinded pathologist are based on a previous study (56). The specific standards are as follows: crypt architecture (normal, 0; severe crypt distortion with loss of entire crypts, 3), degree of inflammatory cell infiltration (normal, 0; dense inflammatory infiltrate, 3), muscle thickening (base of crypt sits on the muscularis mucosae, 0; marked muscle thickening, 3), crypt abscess (absent, 0; present, 1), and goblet cell depletion (absent, 0; present, 1). The total histological scores were the sum of each sample score.

### Sample collection and gut microbe 16S rRNA sequencing analysis

#### 16S rRNA gene amplicon and sequencing

Mice were allowed to empty sterile cages, and the feces were collected immediately in sterile tubes at dry ice and then stored at −80°C. Genomic DNA was isolated using an E.Z.N.A. Soil DNA Kit. DNA concentration was obtained by using Nanodrop 2000 (Thermo Fisher Scientific), and the quality was assessed through agarose gel electrophoresis. The regions V3–V4 of the bacterial 16S rRNA gene were amplified using the primers forward primer (338F 5′-ACTCCTACGGGAGGCAGCA-3′) and reverse primer (806R 5′-GGACTACHVGGGTWTCTAAT-3′). The amplicons were purified and quantified before being sequenced on the Illumina MiSeq platform.

#### Sequence analysis

Data were processed and analyzed using the QIIME (http://www.qiime.org). OTUs were identified with a standard of at least 97% similarity in the 16S rRNA sequence using USEARCH11 (v.7.0.1090, http://drive5.com/uparse/). Beta diversity was measured to reveal the overall distribution difference of microbial community between control and MPTP-treated mice and was visualized in 3D PCA plots. To determine the richness and diversity of each group, alpha diversity calculation was carried out by using four indexes: Sobs, Shannon, Ace, and Chao1. The enterotype analysis of gut microbiota between the two groups was performed according to the methods described previously and shown in PCA plot (21). Venn diagram were generated to display the common and unique microbial species of control and MPTP-treated mice. The bacterial taxonomic distribution at each classification level were analyzed by calculating the relative abundances of OTUs. LEfSe was used to obtain core bacterial groups of the MPTP and control groups. KEGG enzyme and MetaCyc pathway analysis was used to predict the abundances of functional categories by PICRUST2.

### Sample preparation and UHPLC-QE-MS analysis

#### Serum collection

On day 14, mice were sacrificed and the whole blood samples were carefully collected, then kept at room temperature for 1 h to ensure complete clotting and were centrifuged at 5,000 rpm and 4°C for 10 min. The supernatant serum (200 µL) was separated and stored at −80°C until measurement.

#### Metabolite extraction

Fifty microliters of serum samples was transferred to a sterile centrifuge tube, added 200 µL of extract solution (acetonitrile:methanol = 1:1, containing isotopically labeled internal standard mixture), homogenized for 4 min, and sonicated for 10 min in an ice-water bath and incubated for 1 h at −40°C to precipitate proteins. Then, the samples were centrifuged at 12,000 rpm for 5 min. The resulting supernatant was separated to a fresh 2 mL tube for UHPLC-QE-MS analysis. A quality control sample was prepared by mixing an equal aliquot of the supernatant from all samples.

#### UHPLC-QE-MS analysis

UHPLC-QE-MS analysis was performed using an UHPLC System (Vanquish, Thermo Fisher Scientific) with UPLC BEH amide column (2.1 mm × 10.0 mm, 1.7 µm) coupled to Q Exactive HFX mass spectrometer (Orbitrap MS, Thermo). The mobile phase consisted of 25 mmol/L ammonium acetate and 25 mmol/L ammonia hydroxide in water (A) and acetonitrile (B). The auto-sampler temperature was 4°C, and the injection volume was 3 µL. The QE HFX mass spectrometer was employed to acquire Tandem mass spectrometry (MS/MS) spectra based on information-dependent acquisition mode. The detailed parameters are as follows: sheath gas flow rate of 30 Arb, Aux gas flow rate of 25 Arb, capillary temperature of 350°C, full MS resolution of 60,000, MS/MS resolution of 7,500, collision energy of 10/30/60 in NCE mode, and spray Voltage of 3.6 kV (positive) or −3.2 kV (negative), respectively.

#### Data preprocessing and annotation

The raw data were converted to mzXML format by ProteoWizard software. Then the data were processed for peak recognition, peak extraction, peak alignment, integration, and identification based on the R program package (kernel XCMS). Next, PCA was performed to reveal the overall distribution of two groups. The orthogonal partial least square discriminant analysis models were created to display the differences in metabolomics between the two groups. In order to evaluate the metabolite contribution, the value of VIP of each metabolite was calculated. Metabolic pathway enrichment analysis was performed based on KEGG database (http://www.genome.jp/kegg/).

### Statistical analysis

All results were analyzed by the GraphPad Prism v.7 software using two-tailed Student’s *t*-test and Welch’s *t*-test. Pearson’s correlation analysis was used to examine the relationships among microbiota community, microbial metabolomics, and PD-associated features. The data are shown as mean ± SD. Significance was shown as * (*P* value of <0.05), ** (*P* value of <0.01), and *** (*P* value of <0.001).

## Data Availability

All data are included in the manuscript and/or supplemental information files. The 16S rRNA sequencing data have been deposited in the Sequence Read Archive of National Center for Biotechnology Information (Bioproject: PRJNA1012669)

## SUPPLEMENTAL MATERIAL

Table S1 msphere.00431-23-s0001.pdf Distribution of the gut microbiota.

Table S2 msphere.00431-23-s0002.pdf All names of species in the Fig. 3I.

Table S3 msphere.00431-23-s0003.pdf Distribution of serum metabolites.

Table S4 msphere.00431-23-s0004.pdf Pathway analysis of differential metabolites between PD mice and controls.

Table S5 msphere.00431-23-s0005.pdf Pearson's correlation coefficients and *P* values calculated from Spearman correlation analysis between top 50 genera and PD associated results.

Table S6 msphere.00431-23-s0006.pdf Pearson's correlation coefficients and *P* values calculated from Spearman correlation analysis between PD associated results.

ASM does not own the copyrights to Supplemental Material that may be linked to, or accessed through, an article. The authors have granted ASM a non-exclusive, world-wide license to publish the Supplemental Material files. Please contact the corresponding author directly for reuse.
