# Supplementary material for: Gut microbiota-associated taurine metabolism dysregulation in a mouse model of Parkinson’s disease
Source: mSphere. 2023 Oct 11;8(6):e00431-23. doi: 10.1128/msphere.00431-23 (PMC10732050; doi:10.1128/msphere.00431-23)
Supplement: Table S1 — Distribution of the gut microbiota. [file msphere.00431-23-s0001.pdf]

Table S1. Phylum-level distribution of the gut microbiota.

Species name	MPTP-Mean(%)	Control-Mean(%)	P-value	Corrected p-value
p__Firmicutes	81.66	69.11	0.027	0.069
p__Bacteroidetes	1.21	22.70	0.001	0.012
p__Actinobacteria	6.10	3.67	0.091	0.198
p__Proteobacteria	6.05	3.47	0.177	0.288
p__TM7	4.80	0.54	0.020	0.064
p__Tenericutes	0.08	0.19	0.176	0.288
p__Deferribacteres	0.08	0.09	0.847	0.847
p__Verrucomicrobia	0.01	0.11	0.004	0.026

Welch's t-test was used to identify specific bacterial taxa that were dramatically altered (P < 0.05) and p-value was fdr corrected. Taxa that relative abdundance contributed >1% in both groups were included. Significantly changed taxa were marked with asterisk.

Table S1. Class-level distribution of the gut microbiota.

Species name	MPTP-Mean(%]	Control-M	P-value	Corrected p-value
c__Bacilli	61.09	18.65	0.000	0.002
c__Erysipelotrichi	14.61	25.85	0.093	0.237
c__Clostridia	5.96	24.61	0.000	0.000
c__Bacteroidia_p__Bacter	1.21	22.70	0.001	0.007
c__Deltaproteobacteria	5.47	3.02	0.208	0.362
c__Actinobacteria	5.03	1.34	0.012	0.041
c__TM7-3	4.80	0.54	0.020	0.056
c__Coriobacteriia	1.07	2.33	0.011	0.041
c__Gammaproteobacteria	0.31	0.05	0.236	0.362
c__Betaproteobacteria	0.18	0.13	0.513	0.562
c__Mollicutes	0.08	0.17	0.235	0.362
c__Alphaproteobacteria	0.00	0.24	0.154	0.333
c__Deferribacteres	0.08	0.09	0.847	0.885
c__Epsilonproteobacteria	0.09	0.03	0.209	0.362
c__Verrucomicrobiae	0.01	0.11	0.004	0.026
c__unclassified_k__norank	0.00	0.07	0.006	0.028
c__4C0d-2	0.03	0.04	0.499	0.562
c__Chloroplast	0.00	0.00	0.904	0.904

Welch's t-test was used to identify specific bacterial taxa that were dramatically altered ($P < 0.05$) and p-value was fdr corrected. Taxa that relative abdundance contributed >1% in both groups were included. Significantly changed taxa were marked with asterisk.

Table S1. Order-level distribution of the gut microbiota.

Species name	MPTP-Mean(%	Control-Mean(%)	P-value	Corrected p-value
o__Lactobacillales	44.07	18.40	0.002	0.020
o__Erysipelotrichales	14.61	25.85	0.093	0.211
o__Clostridiales	5.96	24.61	0.000	0.000
o__Bacteroidales_c__Bacteroidia	1.21	22.70	0.001	0.015
o__Bacillales	16.35	0.25	0.001	0.017
o__Desulfovibrionales	5.47	3.02	0.208	0.339
o__Bifidobacteriales	5.00	1.34	0.013	0.039
o__CW040	4.80	0.54	0.020	0.055
o__Coriobacteriales	1.07	2.33	0.011	0.037
o__Turicibacterales	0.59	0.00	0.004	0.024
o__Burkholderiales	0.18	0.13	0.513	0.545
o__Pseudomonadales	0.31	0.00	0.183	0.328
o__RF32	0.00	0.23	0.153	0.318
o__RF39	0.07	0.16	0.247	0.351
o__Deferribacterales	0.08	0.09	0.847	0.873
o__Campylobacterales	0.09	0.03	0.209	0.339
o__Verrucomicrobiales	0.01	0.11	0.004	0.025
o__unclassified_k__norank_d__Bacteria	0.00	0.07	0.006	0.029
o__YS2	0.03	0.04	0.499	0.545
o__Enterobacteriales	0.01	0.05	0.070	0.170
o__Gemellales	0.05	0.00	0.330	0.403
o__unclassified_c__Bacilli	0.04	0.00	0.009	0.034
o__Actinomycetales	0.03	0.00	0.049	0.127
o__unclassified_p__Tenericutes	0.00	0.02	0.159	0.318
o__Anaeroplasmatales	0.00	0.01	0.232	0.342
o__Mycoplasmatales	0.01	0.00	0.007	0.030

Welch's t-test was used to identify specific bacterial taxa that were dramatically altered ($P < 0.05$) and p-value was fdr corrected. Taxa that relative abdundance contributed >1% in both groups were included. Significantly changed taxa were marked with asterisk.

Table S1. Family-level distribution of the gut microbiota.

Species name	MPTP-Mean(%)	Control-Mean(%)	P-value	Corrected p-value
f__Erysipelotrichaceae	14.61	25.85	0.093	0.203
f__Aerococcaceae	36.60	0.15	0.000	0.006
f__Lactobacillaceae	7.12	18.04	0.038	0.115
f__S24-7	0.60	20.06	0.001	0.009
f__Lachnospiraceae	1.26	17.32	0.000	0.005
f__Staphylococcaceae	16.34	0.24	0.001	0.015
f__Desulfovibrionaceae	5.47	3.02	0.208	0.319
f__norank_o__Clostridiales	1.78	5.16	0.064	0.175
f__Bifidobacteriaceae	5.00	1.34	0.013	0.056
f__F16	4.80	0.54	0.020	0.077
f__Coriobacteriaceae	1.07	2.33	0.011	0.053
f__Ruminococcaceae	1.86	1.19	0.192	0.306
f__[Paraprevotellaceae]	0.01	1.05	0.070	0.175
f__Peptostreptococcaceae	0.96	0.00	0.036	0.115
f__Rikenellaceae	0.27	0.54	0.223	0.325
f__Turicibacteraceae	0.59	0.00	0.004	0.030
f__Prevotellaceae	0.01	0.54	0.027	0.094
f__unclassified_o__Clostridiales	0.03	0.49	0.010	0.050
f__Streptococcaceae	0.33	0.18	0.366	0.416
f__Bacteroidaceae_o__Bacteroidales	0.09	0.34	0.039	0.115
f__Moraxellaceae	0.31	0.00	0.183	0.299
f__norank_o__RF32	0.00	0.23	0.153	0.278
f__norank_o__RF39	0.07	0.16	0.247	0.345
f__[Mogibacteriaceae]	0.02	0.17	0.001	0.015
f__Deferribacteraceae	0.08	0.09	0.847	0.861
f__[Odoribacteraceae]	0.11	0.05	0.153	0.278
f__Alcaligenaceae	0.04	0.13	0.094	0.203
f__Clostridiaceae	0.04	0.11	0.286	0.383
f__Burkholderiaceae	0.14	0.00	0.037	0.115
f__norank_o__Bacteroidales	0.08	0.04	0.304	0.397
f__Helicobacteraceae	0.09	0.03	0.209	0.319
f__Verrucomicrobiaceae	0.01	0.11	0.004	0.033
f__Dehalobacteriaceae	0.01	0.09	0.088	0.203
f__Porphyromonadaceae	0.02	0.07	0.088	0.203
f__unclassified_k__norank_d__Bacteria	0.00	0.07	0.006	0.040
f__Christensenellaceae	0.00	0.06	0.023	0.084
f__norank_o__YS2	0.03	0.04	0.499	0.548
f__Enterobacteriaceae	0.01	0.05	0.070	0.175
f__Gemellaceae	0.05	0.00	0.330	0.397
f__Enterococcaceae	0.00	0.03	0.000	0.005
f__unclassified_c__Bacilli	0.04	0.00	0.009	0.050
f__unclassified_o__Bacteroidales_c__Bacteroidia	0.02	0.02	0.848	0.861
f__Corynebacteriaceae	0.02	0.00	0.099	0.207
f__Planococcaceae	0.01	0.01	0.609	0.658
f__unclassified_p__Tenericutes	0.00	0.02	0.159	0.280
f__Eubacteriaceae	0.00	0.01	0.002	0.015
f__Anaeroplasmataceae	0.00	0.01	0.232	0.330
f__Mycoplasmataceae	0.01	0.00	0.007	0.042
f__Caulobacteraceae	0.00	0.01	0.223	0.325
f__Carnobacteriaceae	0.01	0.00	0.149	0.278
f__Oxalobacteraceae	0.01	0.00	0.067	0.175
f__norank_c__Alphaproteobacteria	0.00	0.00	0.177	0.299
f__Nocardiaceae	0.00	0.00	0.016	0.067
f__Peptococcaceae	0.00	0.00	0.150	0.278
f__Bacillaceae	0.00	0.00	0.181	0.299
f__Solibacteraceae	0.00	0.00	0.343	0.397
f__norank_o__Streptophyta	0.00	0.00	0.904	0.904
f__Veillonellaceae	0.00	0.00	0.260	0.355
f__Comamonadaceae	0.00	0.00	0.138	0.278
f__Sphingomonadaceae	0.00	0.00	0.446	0.498
f__Microbacteriaceae	0.00	0.00	0.626	0.666
f__Leuconostocaceae	0.00	0.00	0.704	0.737

Welch's t-test was used to identify specific bacterial taxa that were dramatically altered ($P < 0.05$) and p-value was fdr corrected. Taxa that relative abdundance contributed >1% in both groups were included. Significantly changed taxa were marked with asterisk.

Table S1. Genus-level distribution of the gut microbiota.

Species name	MPTP-Mean(%)	Control-Mean(%	P-value	Corrected p-value
g__Allobaculum	14.42	25.73	0.091	0.197
g__Aerococcus	36.55	0.12	0.000	0.016
g__Lactobacillus	7.12	18.04	0.038	0.112
g__norank_f__S24-7	0.60	20.06	0.001	0.020
g__Staphylococcus	15.80	0.12	0.002	0.023
g__Desulfovibrio	5.43	2.97	0.205	0.321
g__norank_f__Lachnospiraceae	0.86	7.30	0.025	0.090
g__norank_o__Clostridiales	1.78	5.16	0.064	0.164
g__unclassified_f__Lachnospiraceae	0.01	6.60	0.011	0.058
g__Bifidobacterium	5.00	1.34	0.013	0.061
g__norank_f__F16	4.80	0.54	0.020	0.077
g__Adlercreutzia	0.67	2.26	0.001	0.023
g__unclassified_f__Ruminococcaceae	1.72	0.08	0.003	0.033
g__Dorea	0.00	1.04	0.025	0.090
g__norank_f__Peptostreptococcaceae	0.96	0.00	0.036	0.112
g__Coprococcus	0.30	0.63	0.134	0.247
g__Oscillospira	0.06	0.70	0.002	0.024
g__norank_f__Rikenellaceae	0.18	0.50	0.105	0.213
g__Jeotgalicoccus	0.53	0.11	0.040	0.112
g__Turicibacter	0.59	0.00	0.004	0.033
g__Prevotella	0.01	0.54	0.027	0.090
g__unclassified_o__Clostridiales	0.03	0.49	0.010	0.056
g__Bacteroides_f__Bacteroidaceae	0.09	0.34	0.039	0.112
g__Streptococcus	0.23	0.18	0.748	0.799
g__norank_f__Coriobacteriaceae	0.32	0.07	0.011	0.056
g__norank_f__Moraxellaceae	0.30	0.00	0.184	0.291
g__norank_f__Ruminococcaceae	0.05	0.19	0.021	0.080
g__norank_o__RF32	0.00	0.23	0.153	0.258
g__norank_o__RF39	0.07	0.16	0.247	0.352
g__Ruminococcus	0.03	0.17	0.003	0.030
g__norank_f__[Mogibacteriaceae]	0.02	0.17	0.001	0.023
g__Mucispirillum	0.08	0.09	0.847	0.880
g__Sutterella	0.04	0.13	0.094	0.197
g__Burkholderia	0.14	0.00	0.037	0.112
g__cc_115	0.12	0.01	0.090	0.197
g__norank_o__Bacteroidales	0.08	0.04	0.304	0.407
g__Odoribacter	0.11	0.00	0.016	0.068
g__Akkermansia	0.01	0.11	0.004	0.036
g__Candidatus_Arthromitus	0.00	0.10	0.112	0.219
g__Roseburia	0.02	0.09	0.144	0.250
g__Lactococcus	0.10	0.00	0.005	0.036
g__Dehalobacterium	0.01	0.09	0.088	0.197
g__Blautia	0.00	0.09	0.033	0.109
g__Parabacteroides	0.02	0.07	0.088	0.197
g__unclassified_f__Coriobacteriaceae	0.08	0.00	0.067	0.168
g__AF12	0.04	0.04	0.908	0.916
g__unclassified_k__norank_d__Bacteria	0.00	0.07	0.006	0.042
g__norank_o__YS2	0.03	0.04	0.499	0.571
g__p-75-a5	0.06	0.00	0.005	0.036
g__[Eubacterium]	0.00	0.06	0.128	0.242
g__Helicobacter	0.03	0.03	0.900	0.916
g__norank_f__Aerococcaceae	0.05	0.01	0.041	0.112
g__norank_f__Desulfovibrionaceae	0.03	0.02	0.441	0.521
g__Rikenella	0.05	0.00	0.014	0.063
g__Butyricimonas	0.00	0.05	0.027	0.090
g__Gemella	0.05	0.00	0.330	0.410
g__Faecalibacterium	0.00	0.04	0.111	0.219
g__Flexispira	0.04	0.00	0.333	0.410
g__Clostridium	0.04	0.00	0.046	0.121
g__norank_f__Erysipelotrichaceae	0.01	0.03	0.010	0.056
g__Enterococcus	0.00	0.03	0.000	0.011
g__unclassified_c__Bacilli	0.04	0.00	0.009	0.055
g__Christensenella	0.00	0.03	0.094	0.197
g__norank_f__Enterobacteriaceae	0.01	0.03	0.141	0.249
g__unclassified_o__Bacteroidales_c__Bacteroidia	0.02	0.02	0.848	0.880
g__Bilophila	0.00	0.03	0.017	0.069
g__norank_f__Christensenellaceae	0.00	0.03	0.085	0.197
g__Facklamia	0.01	0.02	0.304	0.407
g__Corynebacterium	0.02	0.00	0.099	0.203
g__Sporosarcina	0.01	0.01	0.609	0.683
g__unclassified_f__Helicobacteraceae	0.02	0.00	0.271	0.376
g__Coprobacillus	0.01	0.01	0.234	0.342
g__unclassified_f__Erysipelotrichaceae	0.01	0.01	0.484	0.559
g__unclassified_p__Tenericutes	0.00	0.02	0.159	0.264
g__Anaerofustis	0.00	0.01	0.002	0.023
g__Klebsiella	0.00	0.01	0.230	0.342
g__Anaeroplasma	0.00	0.01	0.232	0.342
g__norank_f__Mycoplasmataceae	0.01	0.00	0.007	0.045
g__Paraprevotella	0.01	0.00	0.302	0.407
g__unclassified_f__Caulobacteraceae	0.00	0.01	0.223	0.342
g__Anaerotruncus	0.00	0.00	0.759	0.803
g__Desemzia	0.01	0.00	0.149	0.255

Welch's t-test was used to identify specific bacterial taxa that were dramatically altered ($P < 0.05$) and p-value was fdr corrected. Taxa that relative abdundance contributed >1% in both groups were included.
