# Supplementary material for: Gut microbiota-associated taurine metabolism dysregulation in a mouse model of Parkinson’s disease
Source: mSphere. 2023 Oct 11;8(6):e00431-23. doi: 10.1128/msphere.00431-23 (PMC10732050; doi:10.1128/msphere.00431-23)
Supplement: Table S2 — All names of species in the Fig. 3I. [file msphere.00431-23-s0002.pdf]

Table S2. All names of species in the Figure3I

Species name
a: g__Corynebacterium
b: f__Corynebacteriaceae
c: g__Rhodococcus
d: f__Nocardiaceae
e: o__Actinomycetales
f: g__Bifidobacterium
g: f__Bifidobacteriaceae
h: o__Bifidobacteriales
i: c__Actinobacteria
j: g__Adlercreutzia
k: g__norank_f__Coriobacteriaceae
l: g__unclassified_f__Coriobacteriaceae
m: f__Coriobacteriaceae
n: o__Coriobacteriales
o: c__Coriobacteriia
p: g__Bacteroides_f__Bacteroidaceae
q: f__Bacteroidaceae_o__Bacteroidales
r: g__Parabacteroides
s: f__Porphyromonadaceae
t: g__Prevotella
u: f__Prevotellaceae
v: g__Rikenella
w: g__norank_f__S24-7
x: f__S24-7
y: g__Butyricimonas
z: g__Odoribacter
a0: g__[Prevotella]
a1: f__[Paraprevotellaceae]
a2: o__Bacteroidales_c__Bacteroidia
a3: c__Bacteroidia_p__Bacteroidetes
a4: g__Jeotgalicoccus
a5: g__Staphylococcus
a6: f__Staphylococcaceae
a7: o__Bacillales
a8: g__Aerococcus
a9: f__Aerococcaceae
b0: g__Desemzia
b1: f__Carnobacteriaceae
b2: g__Enterococcus
b3: f__Enterococcaceae
b4: g__Lactobacillus
b5: f__Lactobacillaceae
b6: g__Lactococcus
b7: o__Lactobacillales
b8: g__Turicibacter
b9: f__Turicibacteraceae
c0: o__Turicibacterales
c1: g__unclassified_c__Bacilli
c2: f__unclassified_c__Bacilli
c3: o__unclassified_c__Bacilli
c4: c__Bacilli
c5: g__Christensenella
c6: g__norank_f__Christensenellaceae
c7: f__Christensenellaceae
c8: g__Candidatus_Arthromitus
c9: g__Clostridium
d0: g__Dehalobacterium
d1: f__Dehalobacteriaceae
d2: g__Anaerofustis
d3: f__Eubacteriaceae
d4: g__Anaerostipes
d5: g__Blautia
d6: g__Dorea
d7: g__Roseburia
d8: g__[Ruminococcus]
d9: g__norank_f__Lachnospiraceae
e0: g__unclassified_f__Lachnospiraceae
e1: f__Lachnospiraceae
e2: g__norank_f__Peptostreptococcaceae
e3: f__Peptostreptococcaceae
e4: g__Faecalibacterium
e5: g__Oscillospira
e6: g__Ruminococcus
e7: g__norank_f__Ruminococcaceae
e8: g__unclassified_f__Ruminococcaceae
e9: g__norank_f__[Mogibacteriaceae]
f0: f__[Mogibacteriaceae]
f1: g__unclassified_o__Clostridiales
f2: f__unclassified_o__Clostridiales
f3: o__Clostridiales
f4: c__Clostridia
f5: g__[Eubacterium]
f6: g__cc_115
f7: g__norank_f__Erysipelotrichaceae
f8: g__p-75-a5
f9: g__norank_o__RF32
g0: f__norank_o__RF32
g1: o__RF32
g2: c__Alphaproteobacteria
g3: g__Sutterella
g4: f__Alcaligenaceae
g5: g__Burkholderia
g6: f__Burkholderiaceae
g7: g__Herbaspirillum
g8: g__Bilophila
g9: g__unclassified_f__Helicobacteraceae
h0: g__norank_f__Moraxellaceae
h1: f__Moraxellaceae
h2: o__Pseudomonadales
h3: g__norank_f__F16
h4: f__F16
h5: o__CW040
h6: c__TM7-3
h7: g__norank_f__Mycoplasmataceae
h8: f__Mycoplasmataceae
h9: o__Mycoplasmatales
i0: g__unclassified_p__Tenericutes
i1: f__unclassified_p__Tenericutes
i2: o__unclassified_p__Tenericutes
i3: c__unclassified_p__Tenericutes
i4: g__Akkermansia
i5: f__Verrucomicrobiaceae
i6: o__Verrucomicrobiales
i7: c__Verrucomicrobiae
i8: g__unclassified_k__norank_d__Bacteria
i9: f__unclassified_k__norank_d__Bacteria
j0: o__unclassified_k__norank_d__Bacteria
j1: c__unclassified_k__norank_d__Bacteria

Cladogram of linear discriminant analysis scores for differentially abundant bacteria (p: Phylum, c: Class, o: Order, f: Family). Only taxa meeting an LDA significant threshold of > 2.0 are showed.
