# Supplementary material for: Gut microbiota-associated taurine metabolism dysregulation in a mouse model of Parkinson’s disease
Source: mSphere. 2023 Oct 11;8(6):e00431-23. doi: 10.1128/msphere.00431-23 (PMC10732050; doi:10.1128/msphere.00431-23)
Supplement: Table S3 — Distribution of serum metabolites. [file msphere.00431-23-s0003.pdf]

Table S3. Distribution of serum metabolites.

Name	rt	mz	MPTP-Mean	Control-Mean	VIP	P-VALUE	FOLD CHANGE
Ethyl oleate	42.88	309.28	0.53	0.81	1.63	0.033	0.65
Isopalmitic acid	46.65	255.23	27.45	33.32	1.45	0.047	0.82
L-Norleucine	291.27	130.09	5.02	7.51	1.52	0.039	0.67
Nonadecanoic acid	43.13	297.28	0.08	0.19	1.73	0.010	0.42
Arachidonic acid	44.64	303.23	1.72	2.26	1.66	0.019	0.76
Alpha-Linolenic acid	36.04	277.22	1.02	1.24	1.92	0.005	0.82
2-Hydroxystearic acid	50.18	299.26	0.05	0.09	1.92	0.020	0.59
9,10-epoxyoctadecanoic acid	51.07	297.24	0.25	0.34	1.71	0.020	0.75
Genistein	23.63	269.05	0.14	0.05	1.91	0.006	3.11
Taurocholic acid	199.62	514.28	0.05	0.12	1.74	0.026	0.42
Benzyl acetate	85.71	149.06	2.98	1.73	1.74	0.036	1.73
Mesaconic acid	368.59	129.02	0.46	1.07	2.02	0.002	0.43
Taurine	310.31	124.01	44.04	54.56	1.97	0.004	0.81
Indoleacetaldehyde	42.25	158.06	0.47	0.39	1.50	0.043	1.21
Deoxycytidine	220.51	226.08	0.03	0.04	1.95	0.007	0.74
Creatine	364.20	130.06	0.40	0.63	1.76	0.013	0.64
Ethyl tetradecanoate	36.05	255.23	16.96	20.14	1.63	0.039	0.84
17-HDoHE	49.30	343.23	0.07	0.15	1.91	0.036	0.48
Phenyllactic acid	113.44	165.06	0.87	0.50	1.66	0.029	1.73
2-Pyrocatechuic acid	24.47	153.02	2.00	1.30	1.59	0.047	1.54
Sinapyl alcohol	78.59	209.08	0.06	0.04	1.64	0.020	1.73
3-Sulfinato-L-alaninate	197.32	152.00	0.83	1.64	1.82	0.024	0.51
L-3-Phenyllactic acid	132.22	165.06	0.14	0.08	1.56	0.039	1.78
1H-Indole-2,3-dione	62.99	146.02	0.11	0.17	1.54	0.032	0.64
3-Sulfinoalanine	176.91	152.00	0.07	0.14	1.72	0.025	0.48
Hydroxypyruvic acid	191.30	103.00	0.01	0.02	1.54	0.030	0.72
2-Hydroxyvaleric acid	167.11	117.06	2.96	2.56	1.85	0.008	1.16
Indole-3-propionic acid	93.72	188.07	7.81	3.41	2.24	0.006	2.29
3-Hydroxycapric acid	93.99	187.13	0.66	0.93	1.74	0.033	0.71
Cytidine	254.20	242.08	0.10	0.13	1.95	0.007	0.80
3-Hydroxyisovaleric acid	149.18	117.06	11.80	10.32	1.79	0.010	1.14
Monomethyl glutaric acid	145.62	145.05	0.04	0.02	1.28	0.032	2.73
beta-D-Glucosamine	408.68	214.05	0.02	0.02	1.86	0.007	0.63
(R)-3-Hydroxy-tetradecanoic aci(	63.48	243.20	0.22	0.07	1.96	0.001	2.98
6beta-Hydroxyasiatic acid	176.38	549.34	0.02	0.01	1.94	0.005	3.36

Unpaired two-tailed Student's t test was used to identify specific metabolites that were dramatically altered ($P < 0.05$) . Variable importance of projection (VIP) of each metabolite were obtained based on the orthogonal partial least squares discriminant analysis (OPLS-DA) model. Differential metabolites were identified based on $p < 0.05$ and VIP > 1.
