# Supplementary material for: Gut microbiota-associated taurine metabolism dysregulation in a mouse model of Parkinson’s disease
Source: mSphere. 2023 Oct 11;8(6):e00431-23. doi: 10.1128/msphere.00431-23 (PMC10732050; doi:10.1128/msphere.00431-23)
Supplement: Table S4 — Pathway analysis of differential metabolites between PD mice and controls. [file msphere.00431-23-s0004.pdf]

Table S4. Pathway anlyasis of differential metabolites betwenn PD mice and controls.

Pathway	Impact	Raw p	-ln(p)	Hited metabolites	Counts
Taurine and hypotaurine metabolism*	0.7143	0.000	9.116	3-Sulfinoalanine/Taurine/Taurocholic acid	3.00
Glycine, serine and threonine metabolism	0.0000	0.063	2.767	Hydroxypyruvic acid/Creatine	2.00
Pyrimidine metabolism	0.0097	0.102	2.278	Cytidine/ Deoxycytidine	2.00
Biosynthesis of unsaturated fatty acids	0.0000	0.107	2.237	Arachidonic acid/Alpha-Linolenic acid	2.00
alpha-Linolenic acid metabolism	1.0000	0.115	2.165	Alpha-Linolenic acid	1.00
Primary bile acid biosynthesis	0.0595	0.124	2.085	Taurine/Taurocholic acid	2.00
Glyoxylate and dicarboxylate metabolism	0.0645	0.217	1.528	Hydroxypyruvic acid	1.00
Cysteine and methionine metabolism	0.0253	0.308	1.178	3-Sulfinoalanine	1.00
Arachidonic acid metabolism	0.3260	0.389	0.945	Arachidonic acid	1.00
Tryptophan metabolism	0.0183	0.422	0.864	Indoleacetaldehyde	1.00
Arginine and proline metabolism	0.0120	0.453	0.792	Creatine	1.00

Enrichment analysis was used to identify significant pathways of differental metabolites. The values of pathway impact were obtained based on the topology analysis. The significance of pathway is assessed by the impact value and *p* value.The most significant pathway was marked with asterisk.
