# Supplementary material for: Gut microbiota-associated taurine metabolism dysregulation in a mouse model of Parkinson’s disease
Source: mSphere. 2023 Oct 11;8(6):e00431-23. doi: 10.1128/msphere.00431-23 (PMC10732050; doi:10.1128/msphere.00431-23)
Supplement: Table S5 — Pearson's correlation coefficients and P values calculated from Spearman correlation analysis between top 50 genera and PD associated results. [file msphere.00431-23-s0005.pdf]

Table S5. Pearson's correlation coefficients and p-values calculated from spearman correlation analysis between Top 50 genera and PD associated results.

P-values

Species names	Open Field Test-Total distence	Rotarod Test-Latency time	TH+ cells in SN	TH+ cells in striatum	Fecal pellets at 15min	Pole Test-Descendant time	Iba1+ cell numbers in SN	GFAP+ cell numbers in SN	IL6	TNFA	Colon histopathological
g__Allobaculum	0.6409943	0.317388357	0.470548682	0.26071553	0.162096213	0.092879148	0.44451988	0.247414164	0.457796999	0.259210503	0.057545026
g__Aerococcus	1.05486E-05	0.001814403	0.000361546	0.000222922	0.000285764	0.002426261	1.28156E-05	0.000454162	0.000301316	0.042334568	0.00049254
g__Lactobacillus	0.000427139	0.015795863	6.02895E-05	0.000539941	0.003773052	0.002550415	0.001313248	0.002752663	0.027446731	0.049793497	0.006724319
g__norank_f__S24-7	0.006454781	0.038521761	0.024733234	0.027955597	0.151940368	0.027127014	0.002939682	0.019766762	0.079956082	0.141416568	0.058163179
g__Staphylococcus	0.015437132	0.006482832	0.01795684	0.002077548	0.005148266	0.000182161	0.004986297	0.001653663	0.132517242	0.035292853	0.000411867
g__norank_f__Lachnospiraceae	0.027809965	0.001049982	0.090838832	0.009486437	0.017340384	0.043475375	0.024274843	0.018804588	0.129034136	0.122625845	0.092344776
g__Desulfovibrio	0.647227879	0.410137236	0.957237102	0.56440862	0.214068903	0.704246855	0.480961959	0.474218838	0.313416483	0.951247764	0.237190891
g__norank_o__Clostridiales	0.521969007	0.830609595	0.548179317	0.715491475	0.549629594	0.760877258	0.732070078	0.871714371	0.355884343	0.507136504	0.354124338
g__unclassified_f__Lachnospiraceae	0.029097117	0.066872028	0.092370369	0.047807417	0.034340828	0.047134675	0.016442628	0.013595586	0.11611119	0.145169821	0.064612605
g__Bifidobacterium	0.209695678	0.098599948	0.182182657	0.054611071	0.288073368	0.016181223	0.109525095	0.03455186	0.453526689	0.003383822	0.14484761
g__norank_f__F16	0.103663438	0.046939903	0.143233175	0.066281591	0.064735123	0.037409272	0.0672417	0.041881966	0.709495595	0.602005114	0.272962912
g__Adlercreutzia	1.82502E-05	3.27063E-06	0.003431034	0.000303327	0.002439468	0.004277902	4.19159E-05	0.000267247	0.014666164	0.02288634	0.020083054
g__unclassified_f__Ruminococcaceae	0.009613708	0.02546017	0.044958988	0.058188711	0.24177021	0.080492712	0.036681391	0.034874437	0.261913187	0.021024151	0.308564065
g__Ruminococcus	0.020077344	0.270483862	0.010138735	0.032361051	0.011009759	0.021435753	0.024950357	0.019105155	0.084481879	0.102052498	0.035518059
g__Prevotella	0.109802056	0.783368971	0.03955103	0.200412718	0.245791359	0.130310103	0.141625548	0.189914634	0.256723176	0.282284505	0.18581556
g__Dorea	0.001671937	0.044563423	0.001588871	0.002159071	0.000462111	0.003078842	0.002740807	0.001796937	0.038206527	0.039794763	0.011766493
g__Coprococcus	0.619008368	0.971779509	0.523092568	0.705906731	0.467221983	0.447853459	0.565461301	0.566713719	0.170838104	0.335876243	0.158214638
g__norank_f__Peptostreptococcaceae	0.116669058	0.095216404	0.1349644	0.073373285	0.403906148	0.026648853	0.098917266	0.042475516	0.189046127	1.20678E-05	0.219957554
g__Oscillospira	0.001208528	0.005619765	0.006054049	0.006277801	0.043729959	0.007643155	0.000381503	0.002926578	0.023456439	0.045988256	0.011803171
g__norank_f__Rikenellaceae	0.33712669	0.932407473	0.356941342	0.814123018	0.604308166	0.776468565	0.448882407	0.857030546	0.537272868	0.692982368	0.868086753
g__Jeotgalicoccus	0.021415893	0.009725391	0.042362789	0.01450251	0.012793313	0.010029796	0.01593946	0.011071748	0.541567102	0.398862986	0.018633455
g__Turicibacter	0.002752137	0.050682295	0.008857052	0.025413478	0.057051359	0.153713664	0.007408069	0.040263041	0.031985798	0.300898358	0.164751088
g__unclassified_o__Clostridiales	0.005696513	0.013090648	0.00433314	0.00306365	0.015725062	0.012013227	0.010394223	0.012628911	0.070353874	0.062692131	0.041076106
g__Bacteroides_f__Bacteroidaceae	0.296569352	0.219048957	0.614816381	0.398950712	0.258507015	0.589105721	0.213189753	0.311339872	0.564293269	0.688510755	0.365114165
g__Streptococcus	0.392467882	0.894103135	0.226496609	0.552524245	0.91009494	0.375052047	0.383332418	0.644891609	0.234228563	0.351812167	0.417360105
g__norank_f__Coriobacteriaceae	0.275160216	0.235222195	0.159678507	0.084513211	0.190757885	0.05958987	0.125568574	0.096738494	0.593666344	0.416552207	0.285892987
g__norank_f__Moraxellaceae	0.519039673	0.34157612	0.303547554	0.12801181	0.102467298	0.061971733	0.251961532	0.186335111	0.625753688	0.519837844	0.006189572
g__norank_f__Ruminococcaceae	0.005613015	0.085577608	0.013473014	0.033717903	0.105835876	0.043870574	0.009170793	0.048625226	0.01306865	0.080605968	0.02482866
g__norank_o__RF32	0.02394866	0.176075265	0.020631618	0.088963233	0.231783546	0.054671599	0.018735251	0.062484919	0.133934351	0.178002193	0.069333339
g__norank_o__RF39	0.486691348	0.224646198	0.659578955	0.356962786	0.419360599	0.40563957	0.397773438	0.246622764	0.89282943	0.528225044	0.934547579
g__norank_f__Mogibacteriaceae	0.001966937	0.000922335	0.056631277	0.004793303	0.006717136	0.01110807	0.000570351	0.001099577	0.034346615	0.050798462	0.035986901
g__Sutterella	0.670376834	0.892522188	0.664426883	0.72897534	0.566995478	0.857351512	0.576890607	0.861850477	0.811301762	0.117736812	0.613867518
g__Mucispirillum	0.398298719	0.176665806	0.138697689	0.108783778	0.288985786	0.326384043	0.484228592	0.230864999	0.487982242	0.51864648	0.299934096
g__Burkholderia	0.00670599	0.001714498	0.020619869	0.003612862	0.009825595	0.000664704	0.004050051	0.00105428	0.224348402	0.059241148	0.031213452
g__cc_115	0.397677656	0.277845127	0.212063967	0.086578221	0.080889291	0.041982038	0.176959088	0.133685348	0.570369922	0.45539975	0.004361576
g__Akkermansia	0.001204385	0.002156693	0.011561541	0.00256584	0.059464449	0.006972425	0.000375588	0.002577526	0.042444031	0.057498529	0.038282902
g__norank_o__Bacteroidales	0.424852528	0.204936615	0.468282373	0.183589914	0.331279442	0.084830122	0.298154015	0.098098413	0.298470133	0.002181454	0.395914985
g__Odoribacter	0.139074605	0.093564243	0.073508724	0.019523951	0.037677299	0.013195351	0.037906044	0.02815379	0.320187736	0.320272615	0.040176419
g__Candidatus_Arthromitus	0.056741646	0.024359475	0.038532356	0.021426007	0.09619791	0.071815441	0.073800422	0.072309092	0.200109415	0.184930208	0.136113527
g__Roseburia	0.147586801	0.083405726	0.270896621	0.20691423	0.254480015	0.213536448	0.097625645	0.119553636	0.301599025	0.357854797	0.173409833
g__Dehalobacterium	0.014562799	0.00128432	0.026797829	0.005309775	0.01211194	0.049778177	0.0185327	0.020648766	0.113000154	0.264846023	0.030767347
g__Blautia	0.121132754	0.069367354	0.308078684	0.166557817	0.179140855	0.176848486	0.071163277	0.077802046	0.257782433	0.308876471	0.174218145
g__Lactococcus	0.003525687	0.078517511	0.013040501	0.042162634	0.11800765	0.199204251	0.012403855	0.056990328	0.003974942	0.091292028	0.205597601
g__Parabacteroides	0.671871906	0.987794504	0.65895619	0.931721469	0.793408932	0.857099373	0.636845039	0.904947043	0.927139045	0.076794525	0.68847738
g__unclassified_k__norank_d__Bacteria	0.063098427	0.068050509	0.158996591	0.110555341	0.130098999	0.111155529	0.036287015	0.047876241	0.200686819	0.270743795	0.111749952
g__unclassified_f__Coriobacteriaceae	0.415856478	0.245445273	0.225880169	0.078777076	0.049092426	0.039721672	0.166691255	0.123535715	0.699872991	0.756696963	0.006991732
g__AF12	0.822908348	0.281477077	0.744968333	0.257442393	0.154924908	0.218602932	0.572568365	0.242843279	0.77512156	0.299909554	0.149187311
g__norank_o__YS2	0.364738665	0.284951931	0.273775406	0.196988032	0.313853522	0.182028817	0.225924715	0.207991466	0.712812584	0.68985599	0.628333408
g__Eubacterium	0.145920156	0.06495768	0.399024929	0.185428224	0.201721224	0.206950353	0.084176141	0.088742989	0.283402278	0.33473637	0.217885644
g__p-75-a5	0.026430617	0.017338638	0.025009099	0.006259181	0.124949664	0.001966328	0.012045863	0.003232141	0.235124275	0.001138792	0.108636566

Pearson's correlation coefficients

Species names	Open Field Test-Total distence	Rotarod Test-Latency time	TH+ cells in SN	TH+ cells in striatum	Fecal pellets at 15min	Pole Test-Descendant time	Iba1+ cell numbers in SN	GFAP+ cell numbers in SN	IL6	TNFA	Colon histopathological scores
g__Allobaculum	0.150319373	0.315766383	0.230760791	0.352762337	0.430788024	-0.506502057	-0.244101244	-0.362100204	-0.237252	-0.353805	-0.561357498
g__Aerococcus	-0.931541058	-0.799217665	-0.857577799	-0.871330076	-0.864450999	0.78619862	0.928755173	0.850561673	0.8629337	0.5925294	0.847978575
g__Lactobacillus	0.852484031	0.676058647	0.901993065	0.844995541	0.764617586	-0.783871662	-0.812697877	-0.780259684	-0.632068	-0.576404	-0.732653399
g__norank_f__S24-7	0.735068962	0.60156194	0.640869802	0.630488201	0.440361731	-0.633071375	-0.777095482	-0.658991077	-0.524569	-0.450724	-0.560220069
g__Staphylococcus	-0.677750287	-0.734814081	-0.666425009	-0.793260711	-0.747990303	0.876653082	0.749759301	0.803190299	0.4598826	0.6097233	0.853612638
g__norank_f__Lachnospiraceae	0.630938179	0.821454828	0.509236182	0.711322626	0.669082222	-0.589939569	-0.64242495	-0.662877896	-0.463576	-0.470544	-0.507214199
g__Desulfovibrio	0.147548577	0.262314069	-0.017383672	0.185216766	0.386899973	-0.122603276	-0.22552063	-0.228907852	-0.318232	0.0198208	-0.369479617
g__norank_o__Clostridiales	0.205366429	0.069272978	0.192844863	0.117760197	0.192159457	-0.098427728	-0.110660123	-0.052319245	-0.292695	-0.212572	-0.293719878
g__unclassified_f__Lachnospiraceae	0.627018412	0.545021038	0.507180028	0.58052167	0.612231048	-0.581943582	-0.673078844	-0.686922289	-0.477877	-0.446973	-0.548825827
g__Bifidobacterium	-0.390320611	-0.499045227	-0.412914481	-0.566875516	-0.334388307	0.67427264	0.485572316	0.611670912	0.2394442	0.770153	0.44729297
g__norank_f__F16	-0.492683378	-0.582357921	-0.44890094	-0.546006632	-0.548617172	0.604316771	0.544407013	0.593570617	0.1203398	0.1678751	0.344406318
g__Adlercreutzia	0.923394864	0.946103612	0.769456586	0.862741957	0.785946829	-0.758049745	-0.909088488	-0.86634393	-0.681486	-0.64727	-0.657743619
g__unclassified_f__Ruminococcaceae	-0.710458672	-0.638445886	-0.586642462	-0.56017327	-0.366151375	0.523780464	0.606150965	0.610819416	0.3519353	0.6541148	0.321267077
g__Ruminococcus	0.657766014	0.346079956	0.706982197	0.617609925	0.701499301	-0.65256489	-0.640140473	-0.661648404	-0.51803	-0.494684	-0.609137156
g__Prevotella	0.485242645	0.088959874	0.599063184	0.397728304	0.363259408	-0.462215367	-0.450513769	-0.406363801	-0.355535	-0.33819	-0.409815556
g__Dorea	0.802724128	0.587513932	0.804875682	0.791532912	0.850013088	-0.774839104	-0.780465737	-0.79963595	-0.602337	-0.598478	-0.696991735
g__Coprococcus	0.160169385	-0.011469385	0.20482424	0.121886916	0.232446133	-0.242372403	-0.184725097	-0.184140619	-0.422847	-0.304508	-0.434400783
g__norank_f__Peptostreptococcaceae	-0.477238944	-0.503419264	-0.457325475	-0.53454551	-0.265694476	0.634588119	0.49864021	0.592206783	0.4070913	0.9296278	0.382360191
g__Oscillospira	0.816003249	0.743069096	0.73880447	0.736696574	0.589368197	-0.724929329	-0.855956828	-0.777312115	-0.645255	-0.5844	-0.696778786
g__norank_f__Rikenellaceae	0.303758844	0.027493861	0.292081159	0.07611589	-0.166826679	-0.091857436	-0.241840019	-0.058359733	-0.198024	-0.127478	0.053810047
g__Jeotgalicoccus	-0.652639174	-0.709707535	-0.59246476	-0.682297878	-0.691207124	0.707692322	0.675389419	0.70112156	0.1959794	0.2684497	0.663584369
g__Turicibacter	-0.780268825	-0.574599223	-0.715726922	-0.638600059	-0.562271999	0.438661323	0.726836235	0.597361573	0.6186559	0.3261169	0.42834848
g__unclassified_o__Clostridiales	0.742297807	0.689596517	0.757367411	0.775081609	0.676390264	-0.69556861	-0.705338787	-0.692109575	-0.539328	-0.552133	-0.595444401
g__Bacteroides_f__Bacteroidaceae	0.328886292	0.383055834	0.16206177	0.268401596	0.354293184	-0.173776065	-0.387584196	-0.319527475	-0.185271	0.1294205	-0.28736513
g__Streptococcus	0.271968951	-0.043137961	0.377403985	0.190793669	0.036598131	-0.281704586	-0.277047405	-0.148585943	-0.371653	-0.29507	-0.258427549
g__norank_f__Coriobacteriaceae	-0.342930178	-0.370922174	-0.433031756	-0.517985884	-0.405659451	0.557626655	0.467315312	0.501438909	0.1716842	0.2588606	0.335815076
g__norank_f__Moraxellaceae	-0.206782395	-0.301105229	-0.324433142	-0.464671991	-0.49416717	0.553391513	0.358875529	0.409375456	0.1571342	0.2063962	0.737520896
g__norank_f__Ruminococcaceae	0.743137318	0.51648327	0.687564613	0.613898965	0.490017428	-0.589053617	-0.713503238	-0.578811995	-0.689715	-0.523615	-0.640548692
g__norank_o__RF32	0.643544592	0.418212176	0.655613685	0.511786363	0.373459162	-0.566759612	-0.663163567	-0.552493878	-0.458398	-0.416528	-0.540975947
g__norank_o__RF39	-0.222659683	-0.378797667	-0.142085623	-0.292068711	-0.257357027	0.264751472	0.269047226	0.362664957	-0.043659	0.2023539	-0.026621679
g__norank_f__Mogibacteriaceae	0.795689954	0.826326288	0.563054541	0.751924749	0.732716826	-0.700900947	-0.843187221	-0.819685106	-0.612216	-0.574365	-0.607925389
g__Sutterella	0.137337907	0.043785254	0.139950858	0.111981984	0.184009199	-0.058227521	-0.179410335	-0.056375159	0.0772897	0.4760226	-0.162490775
g__Mucispirillum	0.268759036	0.417694962	0.453481713	0.48645745	0.333793068	-0.310244892	-0.223887558	-0.374140644	-0.222017	-0.206973	-0.326731832
g__Burkholderia	-0.732815336	-0.801653343	-0.655658869	-0.766840657	-0.709039041	0.838014777	0.76093691	0.821298944	0.3790226	0.5582565	0.620836745
g__cc_115	-0.269099796	-0.341135609	-0.388462872	-0.515082213	-0.523200256	0.593339719	0.417438497	0.45865821	0.1824373	0.2384813	0.757018712
g__Akkermansia	0.816138483	0.791582589	0.698190793	0.783588651	0.5578529	-0.730493362	-0.856430247	-0.783375087	-0.592279	-0.561443	-0.602148596
g__norank_o__Bacteroidales	-0.25443103	-0.394092701	-0.231908324	-0.411709549	-0.307275056	0.517537164	0.327869926	0.499687159	0.3276675	0.7910673	0.270068395
g__Odoribacter	-0.45309738	-0.505593076	-0.534334191	-0.659958661	-0.60364778	0.689035803	0.603079513	0.629878529	0.314039	0.3139868	0.597567439
g__Candidatus_Arthromitus	0.562848551	0.642136233	0.601535976	0.65260134	0.502139839	-0.53699562	-0.533879971	-0.536215312	-0.397974	-0.410567	-0.456135087
g__Roseburia	0.4445909	0.519563017	0.345800687	0.392518805	0.357104259	-0.387314167	-0.500294288	-0.473969574	-0.325671	-0.291551	-0.420560268
g__Dehalobacterium	0.681998114	0.813590074	0.634113469	0.746267671	0.695005311	-0.576435723	-0.664002531	-0.655547775	-0.481475	-0.349919	-0.622114117
g__Blautia	0.47220174	0.540920742	0.321572157	0.426702471	0.415538915	-0.417535184	-0.538032158	-0.527769382	-0.354797	-0.321071	-0.419845816
g__Lactococcus	-0.768082031	-0.526699635	-0.689866244	-0.592924015	-0.475715249	0.39870797	0.693359195	0.56238544	0.7619156	0.5086253	0.393565625
g__Parabacteroides	0.136682544	-0.004959856	0.142360237	0.027773472	0.08475465	0.058331381	-0.152168939	0.038701881	0.0296417	0.5292873	-0.129435027
g__unclassified_k__norank_d__Bacteria	0.551427449	0.543071657	0.433668495	0.484348777	0.46243983	-0.483639461	-0.607155623	-0.580376999	-0.397507	-0.345904	-0.482939454
g__unclassified_f__Coriobacteriaceae	-0.259233856	-0.363507207	-0.377867511	-0.526313183	-0.577844355	0.598653402	0.426581351	0.469540398	0.1244934	0.1001951	0.730327699
g__AF12	-0.072466051	-0.338723757	-0.105167455	-0.355033853	-0.437507049	0.383397957	0.181415234	0.365376875	0.0924238	0.3267475	0.443026951
g__norank_o__YS2	-0.287580582	-0.336432779	-0.343859696	-0.400514111	-0.317959568	0.413046554	0.37783399	0.391665337	0.1189119	0.1288359	0.155976521
g__Eubacterium	0.446231081	0.548238845	0.268360927	0.410144156	0.396671613	-0.392490142	-0.518464401	-0.512088196	-0.337452	-0.305192	-0.383949038
g__p-75-a5	-0.63528692	-0.669089845	-0.639943948	-0.736869808	-0.467990234	0.795703616	0.695381997	0.772441568	0.3709941	0.8183288	0.486633697

Top 50 most abundant genus were included for this analysis.
