# Supplementary material for: Gut microbiota-associated taurine metabolism dysregulation in a mouse model of Parkinson’s disease
Source: mSphere. 2023 Oct 11;8(6):e00431-23. doi: 10.1128/msphere.00431-23 (PMC10732050; doi:10.1128/msphere.00431-23)
Supplement: Table S6 — Pearson's correlation coefficients and P values calculated from Spearman correlation analysis between PD associated results. [file msphere.00431-23-s0006.pdf]

Table S6. Pearson's correlation coefficients and p-values calculated from spearman correlation analysis between PD associated results.

Taxon									P-values									Spearman correlation coefficient								
Domain	Kingdom	Phylum	Class	Order	Family	Genus	Species	OTUs	Open Field Tes	Rotarod Test-Lat	TH+ cells ir	TH+ cells ir	Fecal pellet	Pole Test-D	Iba1+ cell r	GFAP+ cell	Colon histo	Open Field	Rotarod Te	TH+ cells ir	TH+ cells ir	Fecal pellet	Pole Test-D	Iba1+ cell r	GFAP+ cell	Colon histopathological scores
d__Bacter	k__norank_d__Bacteria	p__Firmicutes	c__Bacilli	o__Lactobacil	f__Aerococcac	g__Aerococcus	s__norank	OTU463	0.000	0.002	0.000	0.000	0.000	0.002	0.000	0.000	0.000	-0.932	-0.799	-0.858	-0.871	-0.864	0.786	0.929	0.851	0.848
d__Bacter	k__norank_d__Bacteria	p__Firmicutes	c__Erysipelotric	o__Erysipelot	f__Erysipelotri	g__Allobaculum	s__norank	OTU467	0.076	0.031	0.039	0.014	0.008	0.005	0.045	0.018	0.002	0.531	0.621	0.600	0.683	0.723	-0.753	-0.586	-0.667	-0.800
d__Bacter	k__norank_d__Bacteria	p__Firmicutes	c__Bacilli	o__Lactobacil	f__Lactobacilla	g__Lactobacillus	s__norank	OTU401	0.001	0.008	0.000	0.000	0.003	0.002	0.001	0.002	0.010	0.845	0.723	0.886	0.870	0.776	-0.802	-0.815	-0.806	-0.707
d__Bacter	k__norank_d__Bacteria	p__Firmicutes	c__Bacilli	o__Bacillales	f__Staphylococ	g__Staphylococcus	s__sciuri	OTU482	0.050	0.022	0.040	0.006	0.015	0.000	0.016	0.005	0.001	-0.577	-0.652	-0.599	-0.741	-0.678	0.850	0.675	0.746	0.845
d__Bacter	k__norank_d__Bacteria	p__Firmicutes	c__Clostridia	o__Clostridial	f__Lachnospira	g__unclassified_f__Lachnospiraceae	s__unclas	OTU46	0.034	0.073	0.104	0.054	0.039	0.053	0.019	0.016	0.071	0.614	0.536	0.493	0.567	0.600	-0.569	-0.661	-0.675	-0.538
d__Bacter	k__norank_d__Bacteria	p__Actinobacte	c__Actinobacter	o__Bifidobact	f__Bifidobacte	g__Bifidobacterium	s__pseudo	OTU405	0.210	0.099	0.182	0.055	0.288	0.016	0.109	0.035	0.145	-0.390	-0.499	-0.413	-0.567	-0.335	0.674	0.486	0.612	0.447
d__Bacter	k__norank_d__Bacteria	p__Firmicutes	c__Clostridia	o__Clostridial	f__Lachnospira	g__norank_f__Lachnospiraceae	s__norank	OTU282	0.015	0.000	0.100	0.006	0.010	0.028	0.009	0.006	0.070	0.678	0.883	0.498	0.743	0.710	-0.630	-0.715	-0.737	-0.540
d__Bacter	k__norank_d__Bacteria	p__Firmicutes	c__Bacilli	o__Bacillales	f__Staphylococ	g__Staphylococcus	s__norank	OTU451	0.005	0.005	0.021	0.010	0.008	0.011	0.007	0.007	0.027	-0.749	-0.745	-0.655	-0.706	-0.722	0.700	0.734	0.728	0.634
d__Bacter	k__norank_d__Bacteria	p__Firmicutes	c__Bacilli	o__Lactobacil	f__Lactobacilla	g__Lactobacillus	s__norank	OTU470	0.002	0.055	0.001	0.011	0.051	0.009	0.002	0.012	0.024	0.796	0.567	0.820	0.700	0.575	-0.718	-0.805	-0.694	-0.643
d__Bacter	k__norank_d__Bacteria	p__Bacteroidet	c__Bacteroidia_	o__Bacteroida	f__S24-7	g__norank_f__S24-7	s__norank	OTU75	0.004	0.010	0.031	0.011	0.067	0.015	0.001	0.005	0.043	0.764	0.710	0.622	0.702	0.545	-0.682	-0.818	-0.753	-0.590
d__Bacter	k__norank_d__Bacteria	p__Firmicutes	c__Erysipelotric	o__Erysipelot	f__Erysipelotri	g__Allobaculum	s__norank	OTU122	0.084	0.352	0.131	0.312	0.468	0.771	0.209	0.409	0.766	-0.519	-0.295	-0.462	-0.319	-0.232	0.094	0.391	0.263	0.096
d__Bacter	k__norank_d__Bacteria	p__Proteobacte	c__Deltaproteol	o__Desulfovib	f__Desulfovibr	g__Desulfovibrio	s__norank	OTU398	0.024	0.006	0.148	0.021	0.026	0.047	0.012	0.007	0.084	0.644	0.743	0.445	0.653	0.637	-0.582	-0.698	-0.730	-0.518
d__Bacter	k__norank_d__Bacteria	p__TM7	c__TM7-3	o__CW040	f__F16	g__norank_f__F16	s__norank	OTU471	0.104	0.047	0.143	0.066	0.065	0.037	0.067	0.042	0.273	-0.493	-0.582	-0.449	-0.546	-0.549	0.604	0.544	0.594	0.344
d__Bacter	k__norank_d__Bacteria	p__Proteobacte	c__Deltaproteol	o__Desulfovib	f__Desulfovibr	g__Desulfovibrio	s__C21_c2	OTU500	0.025	0.033	0.040	0.039	0.321	0.043	0.037	0.024	0.568	-0.639	-0.615	-0.599	-0.600	-0.313	0.591	0.606	0.643	0.183
d__Bacter	k__norank_d__Bacteria	p__Bacteroidet	c__Bacteroidia_	o__Bacteroida	f__S24-7	g__norank_f__S24-7	s__norank	OTU223	0.038	0.155	0.084	0.120	0.408	0.086	0.022	0.091	0.170	0.604	0.438	0.519	0.473	0.263	-0.515	-0.649	-0.510	-0.423
d__Bacter	k__norank_d__Bacteria	p__Firmicutes	c__Clostridia	o__Clostridial	f__Lachnospira	g__norank_f__Lachnospiraceae	s__norank	OTU206	0.145	0.033	0.165	0.064	0.108	0.163	0.166	0.140	0.242	0.447	0.616	0.428	0.550	0.487	-0.430	-0.428	-0.452	-0.366
d__Bacter	k__norank_d__Bacteria	p__Firmicutes	c__Bacilli	o__Lactobacil	f__Lactobacilla	g__Lactobacillus	s__norank	OTU447	0.289	0.774	0.147	0.543	0.397	0.564	0.398	0.598	0.438	0.334	-0.093	0.445	0.195	0.269	-0.186	-0.269	-0.170	-0.247
d__Bacter	k__norank_d__Bacteria	p__Bacteroidet	c__Bacteroidia_	o__Bacteroida	f__S24-7	g__norank_f__S24-7	s__norank	OTU515	0.019	0.067	0.050	0.071	0.320	0.056	0.010	0.054	0.108	0.663	0.545	0.576	0.538	0.314	-0.565	-0.711	-0.568	-0.487
d__Bacter	k__norank_d__Bacteria	p__Firmicutes	c__Bacilli	o__Lactobacil	f__Lactobacilla	g__Lactobacillus	s__reuteri	OTU460	0.029	0.059	0.009	0.012	0.020	0.040	0.044	0.049	0.026	0.627	0.558	0.712	0.694	0.657	-0.597	-0.588	-0.578	-0.636
d__Bacter	k__norank_d__Bacteria	p__Firmicutes	c__Clostridia	o__Clostridial	f__Lachnospiraceae				0.055	0.471	0.026	0.082	0.037	0.053	0.064	0.050	0.069	0.567	0.231	0.637	0.521	0.605	-0.569	-0.549	-0.576	-0.541
d__Bacter	k__norank_d__Bacteria	p__Firmicutes	c__Clostridia	o__Clostridial	f__norank_o__	g__norank_o__Clostridiales	s__norank	OTU473	0.940	0.843	0.842	0.889	0.892	0.769	0.876	0.748	0.672	0.025	0.064	-0.065	-0.045	0.044	0.095	0.051	0.104	-0.136
d__Bacter	k__norank_d__Bacteria	p__Firmicutes	c__Clostridia	o__Clostridial	f__Ruminococ	g__unclassified_f__Ruminococcaceae	s__unclas	OTU408	0.009	0.024	0.044	0.056	0.238	0.078	0.034	0.033	0.301	-0.715	-0.642	-0.590	-0.564	-0.369	0.528	0.612	0.615	0.326
d__Bacter	k__norank_d__Bacteria	p__Actinobacte	c__Coriobacterii	o__Coriobacte	f__Coriobacter	g__Adlercreutzia	s__norank	OTU440	0.000	0.000	0.005	0.000	0.002	0.002	0.000	0.000	0.009	0.881	0.940	0.754	0.863	0.801	-0.798	-0.903	-0.858	-0.717
d__Bacter	k__norank_d__Bacteria	p__Firmicutes	c__Erysipelotric	o__Erysipelot	f__Erysipelotri	g__Allobaculum	s__norank	OTU402	0.039	0.137	0.067	0.116	0.240	0.332	0.064	0.135	0.604	-0.600	-0.455	-0.545	-0.478	-0.367	0.307	0.549	0.457	0.167
d__Bacter	k__norank_d__Bacteria	p__Firmicutes	c__Clostridia	o__Clostridial	f__Lachnospira	g__norank_f__Lachnospiraceae	s__norank	OTU53	0.112	0.022	0.223	0.071	0.086	0.147	0.114	0.127	0.248	0.483	0.650	0.380	0.538	0.515	-0.445	-0.480	-0.465	-0.362
d__Bacter	k__norank_d__Bacteria	p__Bacteroidet	c__Bacteroidia_	o__Bacteroida	f__S24-7	g__norank_f__S24-7	s__norank	OTU304	0.018	0.103	0.055	0.061	0.239	0.047	0.010	0.042	0.121	0.664	0.494	0.566	0.554	0.368	-0.581	-0.708	-0.594	-0.473
d__Bacter	k__norank_d__Bacteria	p__Bacteroidet	c__Bacteroidia_	o__Bacteroida	f__S24-7	g__norank_f__S24-7	s__norank	OTU171	0.004	0.007	0.052	0.011	0.022	0.028	0.002	0.006	0.042	0.756	0.727	0.573	0.702	0.652	-0.631	-0.807	-0.737	-0.593
d__Bacter	k__norank_d__Bacteria	p__Firmicutes	c__Clostridia	o__Clostridial	f__Lachnospira	g__Coprococcus	s__norank	OTU446	0.776	0.786	0.626	0.854	0.580	0.561	0.733	0.729	0.212	0.092	-0.088	0.157	0.060	0.178	-0.187	-0.110	-0.112	-0.388
d__Bacter	k__norank_d__Bacteria	p__Firmicutes	c__Bacilli	o__Lactobacil	f__Lactobacilla	g__Lactobacillus	s__unclas	OTU79	0.416	0.362	0.384	0.289	0.458	0.292	0.290	0.262	0.919	-0.259	-0.289	-0.277	-0.334	-0.237	0.332	0.333	0.352	0.033
d__Bacter	k__norank_d__Bacteria	p__Bacteroidet	c__Bacteroidia_	o__Bacteroida	f__S24-7	g__norank_f__S24-7	s__norank	OTU449	0.013	0.077	0.008	0.028	0.244	0.037	0.015	0.060	0.088	0.689	0.529	0.725	0.629	0.365	-0.606	-0.682	-0.557	-0.513
d__Bacter	k__norank_d__Bacteria	p__Bacteroidet	c__Bacteroidia_	o__Bacteroida	f__S24-7	g__norank_f__S24-7	s__norank	OTU419	0.007	0.038	0.018	0.030	0.191	0.025	0.003	0.023	0.057	0.734	0.603	0.666	0.625	0.405	-0.639	-0.775	-0.646	-0.562
d__Bacter	k__norank_d__Bacteria	p__Firmicutes	c__Bacilli	o__Bacillales	f__Staphylococ	g__Staphylococcus	s__unclas	OTU486	0.005	0.004	0.012	0.002	0.004	0.001	0.003	0.002	0.001	-0.748	-0.768	-0.696	-0.789	-0.761	0.836	0.779	0.805	0.812
d__Bacter	k__norank_d__Bacteria	p__Bacteroidet	c__Bacteroidia_	o__Bacteroida	f__S24-7	g__norank_f__S24-7	s__norank	OTU292	0.035	0.194	0.049	0.118	0.440	0.082	0.026	0.109	0.156	0.610	0.403	0.577	0.475	0.247	-0.522	-0.638	-0.487	-0.436
d__Bacter	k__norank_d__Bacteria	p__Firmicutes	c__Clostridia	o__Clostridial	f__norank_o__	g__norank_o__Clostridiales	s__norank	OTU324	0.021	0.248	0.004	0.031	0.073	0.029	0.034	0.056	0.070	0.654	0.362	0.758	0.621	0.536	-0.627	-0.613	-0.565	-0.540
d__Bacter	k__norank_d__Bacteria	p__Bacteroidet	c__Bacteroidia_	o__Bacteroida	f__S24-7	g__norank_f__S24-7	s__norank	OTU286	0.020	0.112	0.055	0.044	0.042	0.035	0.012	0.014	0.061	0.658	0.483	0.567	0.590	0.593	-0.610	-0.695	-0.687	-0.555
d__Bacter	k__norank_d__Bacteria	p__Firmicutes	c__Bacilli	o__Bacillales	f__Staphylococ	g__Jeotgalicoccus	s__psychr	OTU474	0.021	0.010	0.042	0.015	0.013	0.010	0.016	0.011	0.019	-0.653	-0.710	-0.592	-0.682	-0.691	0.708	0.675	0.701	0.664
d__Bacter	k__norank_d__Bacteria	p__Firmicutes	c__Clostridia	o__Clostridial	f__Peptostrept	g__norank_f__Peptostreptococcaceae	s__norank	OTU498	0.117	0.095	0.135	0.073	0.404	0.027	0.099	0.042	0.220	-0.477	-0.503	-0.457	-0.535	-0.266	0.635	0.499	0.592	0.382
d__Bacter	k__norank_d__Bacteria	p__Actinobacte	c__Coriobacterii	o__Coriobacte	f__Coriobacter	g__Adlercreutzia	s__norank	OTU491	0.002	0.000	0.021	0.001	0.004	0.006	0.001	0.001	0.019	0.800	0.948	0.653	0.825	0.763	-0.735	-0.832	-0.815	-0.662
d__Bacter	k__norank_d__Bacteria	p__Firmicutes	c__Bacilli	o__Turicibact	f__Turicibacter	g__Turicibacter	s__norank	OTU495	0.003	0.051	0.009	0.026	0.058	0.155	0.008	0.041	0.167	-0.780	-0.574	-0.715	-0.637	-0.561	0.437	0.726	0.596	0.426
d__Bacter	k__norank_d__Bacteria	p__Proteobacte	c__Gammaprote	o__Pseudomo	f__Moraxellace	g__norank_f__Moraxellaceae	s__norank	OTU503	0.519	0.342	0.304	0.128	0.102	0.062	0.252	0.186	0.006	-0.207	-0.301	-0.324	-0.465	-0.494	0.553	0.359	0.409	0.738
d__Bacter	k__norank_d__Bacteria	p__Bacteroidet	c__Bacteroidia_	o__Bacteroida	f__S24-7	g__norank_f__S24-7	s__norank	OTU513	0.117	0.253	0.129	0.273	0.440	0.343	0.093	0.316	0.173	0.477	0.358	0.463	0.344	0.247	-0.301	-0.506	-0.317	-0.421
d__Bacter	k__norank_d__Bacteria	p__Actinobacte	c__Coriobacterii	o__Coriobacte	f__Coriobacter	g__Adlercreutzia	s__norank	OTU228	0.220	0.335	0.302	0.465	0.720	0.859	0.490	0.499	0.614	0.382	0.305	0.325	0.234	0.116	-0.058	-0.221	-0.217	0.162
d__Bacter	k__norank_d__Bacteria	p__Actinobacte	c__Coriobacterii	o__Coriobacte	f__Coriobacter	g__Adlercreutzia	s__norank	OTU429	0.000	0.024	0.001	0.001	0.006	0.002	0.000	0.001	0.017	0.861	0.645	0.818	0.821	0.742	-0.789	-0.860	-0.841	-0.669
d__Bacter	k__norank_d__Bacteria	p__Bacteroidet	c__Bacteroidia_	o__Bacteroida	f__S24-7	g__norank_f__S24-7	s__norank	OTU532	0.000	0.000	0.002	0.000	0.018	0.003	0.000	0.000	0.008	0.859	0.881	0.798	0.854	0.666	-0.773	-0.894	-0.851	-0.723
d__Bacter	k__norank_d__Bacteria	p__Firmicutes	c__Erysipelotric	o__Erysipelot	f__Erysipelotri	g__Allobaculum	s__norank	OTU44	0.125	0.388	0.173	0.356	0.566	0.782	0.265	0.443	0.875	-0.468	-0.275	-0.421	-0.293	-0.184	0.090	0.350	0.245	0.051
d__Bacter	k__norank_d__Bacteria	p__Firmicutes	c__Clostridia	o__Clostridial	f__norank_o__	g__norank_o__Clostridiales	s__norank	OTU455	0.003	0.006	0.016	0.011	0.031	0.044	0.007	0.028	0.098	0.770	0.740	0.677	0.704	0.620	-0.590	-0.728	-0.631	-0.499
d__Bacter	k__norank_d__Bacteria	p__Firmicutes	c__Clostridia	o__Clostridial	f__norank_o__	g__norank_o__Clostridiales	s__norank	OTU168	0.107	0.787	0.032	0.188	0.195	0.157	0.175	0.196	0.242	0.489	0.088	0.619	0.408	0.402	-0.436	-0.419	-0.401	-0.366
d__Bacter	k__norank_d__Bacteria	p__Bacteroidet	c__Bacteroidia_	o__Bacteroida	f__S24-7	g__norank_f__S24-7	s__norank	OTU188	0.007	0.043	0.041	0.019	0.056	0.019	0.003	0.007	0.062	0.729	0.592	0.594	0.661	0.565	-0.663	-0.776	-0.728	-0.553
d__Bacter	k__norank_d__Bacteria	p__Firmicutes	c__Clostridia	o__Clostridial	f__Ruminococ	g__Oscillospira	s__norank	OTU166	0.030	0.004	0.108	0.034	0.071	0.058	0.014	0.018	0.063	0.623	0.759	0.487	0.614	0.538	-0.560	-0.685	-0.668	-0.552
d__Bacter	k__norank_d__Bacteria	p__Firmicutes	c__Clostridia	o__Clostridial	f__Lachnospira	g__norank_f__Lachnospiraceae	s__norank	OTU395	0.008	0.002	0.005	0.000	0.005	0.009	0.010	0.002	0.025	0.726	0.804	0.753	0.863	0.754	-0.714	-0.708	-0.794	-0.640
d__Bacter	k__norank_d__Bacteria	p__Bacteroidet	c__Bacteroidia_	o__Bacteroida	f__S24-7	g__norank_f__S24-7	s__norank	OTU9	0.007	0.023	0.013	0.020	0.088	0.021	0.003	0.010	0.027	0.732	0.648	0.688	0.658	0.514	-0.656	-0.770	-0.708	-0.634
d__Bacter	k__norank_d__Bacteria	p__Bacteroidet	c__Bacteroidia_	o__Bacteroida	f__S24-7	g__norank_f__S24-7	s__norank	OTU297	0.002	0.001	0.003	0.001	0.024	0.008	0.002	0.005	0.022	0.790	0.814	0.775	0.831	0.642	-0.724	-0.801	-0.745	-0.651
d__Bacter	k__norank_d__Bacteria	p__Firmicutes	c__Bacilli	o__Lactobacil	f__Streptococc	g__Streptococcus	s__norank	OTU493	0.376	0.913	0.213	0.533	0.891	0.362	0.367	0.627	0.399	0.281	-0.035	0.387	0.200	0.045	-0.289	-0.286	-0.157	-0.268
d__Bacter	k__norank_d__Bacteria	p__Actinobacte	c__Coriobacterii	o__Coriobacte	f__Coriobacter	g__Adlercreutzia	g__norank	OTU497	0.092	0.062	0.054	0.015	0.046	0.010	0.026	0.017	0.081	-0.508	-0.553	-0.568	-0.682	-0.585	0.710	0.636	0.671	0.523
d__Bacter	k__norank_d__Bacteria	p__Bacteroidet	c__Bacteroidia_	o__Bacteroida	f__S24-7	g__norank_f__S24-7	s__norank	OTU340	0.005	0.008	0.028	0.014	0.059	0.018	0.002	0.005	0.029	0.750	0.722	0.631	0.687	0.558	-0.665	-0.800	-0.746	-0.628
d__Bacter	k__norank_d__Bacteria	p__Bacteroidet	c__Bacteroidia_	o__Bacteroida	f__Rikenellace	g__norank_f__Rikenellaceae	s__norank	OTU414	0.820	0.600	0.991	0.484	0.183	0.412	0.869	0.522	0.126	0.074	-0.169	0.003	-0.224	-0.412	0.261	0.054	0.205	0.467
d__Bacter	k__norank_d__Bacteria	p__Bacteroidet	c__Bacteroidia_	o__Bacteroida	f__S24-7	g__norank_f__S24-7	s__norank	OTU385	0.039	0.289	0.019	0.092	0.385	0.068	0.041	0.112	0.144	0.600	0.334	0.661	0.508	0.276	-0.543	-0.596	-0.483	-0.448
d__Bacter	k__norank_d__Bacteria	p__Bacteroidet	c__Bacteroidia_	o__Bacteroida	f__S24-7	g__norank_f__S24-7	s__norank	OTU236	0.879	0.700	0.867	0.602	0.861	0.529	0.786	0.524	0.537	-0.049	-0.124	-0.054	-0.168	-0.057	0.202	0.088	0.205	-0.198
d__Bacter	k__norank_d__Bacteria	p__Actinobacte	c__Coriobacterii	o__Coriobacte	f__Coriobacter	g__Adlercreutzia	s__norank	OTU485	0.001	0.002	0.019	0.002	0.010	0.007	0.001	0.000	0.037	0.811	0.793	0.663	0.806	0.706	-0.728	-0.842	-0.851	-0.605
d__Bacter	k__norank_d__Bacteria	p__Firmicutes	c__Erysipelotric	o__Erysipelot	f__Erysipelotri	g__Allobaculum	s__norank	OTU123	0.055	0.170	0.091	0.163	0.331	0.425	0.100	0.187	0.696	-0.566	-0.424	-0.509	-0.430	-0.308	0.254	0.497	0.408	0.126
d__Bacter	k__norank_d__Bacteria	p__Actinobacte	c__Coriobacterii	o__Coriobacte	f__Coriobacter	g__Adlercreutzia	s__norank	OTU437	0.001	0.000	0.015	0.002	0.008	0.007	0.000	0.002	0.021	0.828	0.940	0.678	0.802	0.719	-0.728	-0.858	-0.804	-0.653

OTUs contributing > 0.1% are included for this analysis.
